# Overexpression of a Cytochrome P450 Monooxygenase Involved in Orobanchol Biosynthesis Increases Susceptibility to Fusarium Head Blight

**DOI:** 10.3389/fpls.2021.662025

**Published:** 2021-04-01

**Authors:** Valentin Changenet, Catherine Macadré, Stéphanie Boutet-Mercey, Kévin Magne, Mélanie Januario, Marion Dalmais, Abdelhafid Bendahmane, Grégory Mouille, Marie Dufresne

**Affiliations:** ^1^Université Paris-Saclay, CNRS, INRAE, University of Evry, Institute of Plant Sciences Paris-Saclay, Orsay, France; ^2^Université de Paris, Institute of Plant Sciences Paris-Saclay, Orsay, France; ^3^Institut Jean-Pierre Bourgin, INRAE, AgroParisTech, Université Paris-Saclay, Versailles, France

**Keywords:** *Brachypodium distachyon*, Fusarium Head Blight, cytochrome P450, orobanchol, susceptibility

## Abstract

Fusarium Head Blight (FHB) is a cereal disease caused primarily by the ascomycete fungus *Fusarium graminearum* with public health issues due to the production of mycotoxins including deoxynivalenol (DON). Genetic resistance is an efficient protection means and numerous quantitative trait loci have been identified, some of them related to the production of resistance metabolites. In this study, we have functionally characterized the *Brachypodium distachyon BdCYP711A29* gene encoding a cytochrome P450 monooxygenase (CYP). We showed that *BdCYP711A29* belongs to an oligogenic family of five members. However, following infection by *F. graminearum*, *BdCYP711A29* is the only copy strongly transcriptionally induced in a DON-dependent manner. The BdCYP711A29 protein is homologous to the *Arabidopsis thaliana* MAX1 and *Oryza sativa* MAX1-like CYPs representing key components of the strigolactone biosynthesis. We show that BdCYP711A29 is likely involved in orobanchol biosynthesis. Alteration of the *BdCYP711A29* sequence or expression alone does not modify plant architecture, most likely because of functional redundancy with the other copies. *B. distachyon* lines overexpressing *BdCYP711A29* exhibit an increased susceptibility to *F. graminearum*, although no significant changes in defense gene expression were detected. We demonstrate that both orobanchol and exudates of *Bd711A29* overexpressing lines stimulate the germination of *F. graminearum* macroconidia. We therefore hypothesize that orobanchol is a susceptibility factor to FHB.

## Introduction

Fusarium Head Blight (FHB) is a major disease of small-grain cereals, including wheat (Dweba et al., 2017; Figueroa et al., 2017; Duba et al., 2018). FHB is due to a complex association of different ascomycete fungal species belonging to either the *Fusarium* or the *Microdochium* genera (Siou et al., 2015) but is primarily due to *Fusarium graminearum* (teleomorph *Gibberella zeae*) (Goswami and Kistler, 2004). FHB damages consist of yield losses and in contamination of grains by mycotoxins which are harmful for humans and animals (Yazar and Omurtag, 2008; McCormick et al., 2011). *F. graminearum* has indeed the ability to produce secondary metabolites mainly belonging to type B trichothecenes, including deoxynivalenol (DON), representing potent inhibitors of eukaryotic translation (Rocha et al., 2005).

Cereals protection toward FHB has for a long time relied on chemicals mainly belonging to the demethylation inhibitors (DMI) family, however none confers full protection against the disease (Yuen and Schoneweis, 2007; Dweba et al., 2017; Chen et al., 2019). Alternative strategies such as biocontrol (Comby et al., 2017; Legrand et al., 2017) or innovative strategies using interfering RNAs (Machado et al., 2017; He et al., 2019) are emerging but they are not fully operational yet. Cultivar resistance remains therefore the most efficient protection strategy. Resistance to FHB is polygenic and more than 300 QTLs involved in partial resistance to the disease have been identified in the wheat genome (recently reviewed in Venske et al., 2019). Numerous large-scale studies have been performed to decipher the biological functions associated with resistance to FHB. These were designed to identify either differentially expressed genes (DEGs; for a review, see Kazan and Gardiner, 2017) or differentially produced metabolites (Kumaraswamy et al., 2011; Gunnaiah et al., 2012; Gauthier et al., 2015) related to quantitative resistance. Although these results could not be properly compared due to diverse host genetic backgrounds, they nevertheless allowed the identification of resistance-related families of secondary metabolites: phenylpropanoids reinforcing cell walls or scavenging reactive oxygen species, lignins, and lignans involved in cell wall thickness, polyamines strengthening physical barriers through their ability to bind cell wall components, terpenoids often having antimicrobial activities, and fatty acids which oxidation products can lead among others to jasmonate (JA) precursors (Bollina et al., 2010, 2011; Kumaraswamy et al., 2011; Kushalappa and Gunnaiah, 2013; Gunnaiah and Kushalappa, 2014; Kage et al., 2017; Karre et al., 2017).

Jasmonates are well-known phytohormones involved in plant responses to biotic stress, and are especially associated with defense against necrotrophs and herbivorous pests (Yang et al., 2013; Wasternack and Song, 2017). *F. graminearum*, the causal agent of FHB has been described as a hemibiotroph through deep cytological studies (Brown et al., 2010). JA are therefore not likely the only phytohormones involved in cereals defense against FHB. Recent works have explored the role and the possible involvement of different phytohormones in FHB-induced resistance. Salicylic acid (SA) was shown in several studies to promote basal resistance to FHB during early infection stages (Makandar et al., 2010, 2012; Ding et al., 2011). However, these studies mostly relied on the analysis of DEGs during infection of cereal cultivars exhibiting contrasting response to the disease. Nevertheless, in a very recent work, Qi et al. (2019) have demonstrated by using a transgenic *F. graminearum* strain able to metabolize SA, that endogenous SA levels influence the resistance to FHB. Numerous studies have concluded on the role of ethylene (ET) in the promotion of FHB resistance at later infection stages (Makandar et al., 2010; Ding et al., 2011; Gottwald et al., 2012; Sun et al., 2016; Wang et al., 2018b). In contrast, other researches have shown that ET promotes disease (Chen et al., 2009) or has no impact on disease development (Sun et al., 2016). Other phytohormones less frequently associated with plant responses to biotic stress may also play a role in infection. Abscisic acid (ABA) was shown to increase susceptibility whereas gibberellic acids promote FHB resistance but these effects appeared mostly due to the modulation of *F. graminearum* gene expression of Buhrow et al. (2016). Brassinosteroids (BR) were also shown to improve FHB resistance either following exogenous application of epibrassinolide (Ali et al., 2013) or by using *Brachypodium distachyon* or barley mutants insensitive to BR (Chen et al., 2014; Goddard et al., 2014). A recent integrated transcriptome and hormone profiling study has investigated the involvement and modulation of five major phytohormones, SA, JA, ET, ABA, and auxin, during FHB infection. Wheat cultivars exhibiting contrasting responses to FHB were used including the well-known highly resistant Sumai 3 accession (Wang et al., 2018a). This work has indicated that the Sumai3 resistance is likely due to elevated basal SA levels coupled with stronger and faster production of ET. In contrast, susceptible varieties also showed increased ET levels but at late stages of infection during which ET may rather promote disease spread (Wang et al., 2018a). This study, as several previous ones in other plant species, therefore confirmed the sequential roles of the phytohormones at different infection stages and also points out the complexity of possible crosstalks in defense against biotic stress (Kazan, 2013; Yang et al., 2013; Pozo et al., 2015; Akamatsu et al., 2016).

Strigolactones (SLs) constitute a new class of phytohormones. SLs are carotenoid-derived compounds for which both the biosynthetic pathway and the perception have been extensively studied in the last decade (de Saint Germain et al., 2013; Ruyter-Spira et al., 2013; Lopez-Obando et al., 2015; Marzec, 2016). The SLs biosynthesis can be divided into two parts: the core and the diversification biosynthetic pathways. The core pathway is highly conserved among plant species and starts from all-*trans*-β-carotene. It involves activities of three successive enzymes—namely an all-trans-/9-cis-β-carotene isomerase and two carotenoid-cleavage dioxygenases, to end up with the formation of carlactone (CL; Ruyter-Spira et al., 2013). The diversification pathway is named after the high diversity of SLs it can produce. It is less conserved and involves cytochrome P450 monooxygenases (CYP) belonging to the CYP711A subfamily (Nelson, 2009; Wang and Bouwmeester, 2018). The first functionally characterized member of the CYP711A subfamily was the *Arabidopsis thaliana* MAX1 enzyme (Booker et al., 2005). Later studies have led to the identification of MAX1 homologs in various plant species such as petunia (Drummond et al., 2012), poplar (Czarnecki et al., 2014), rice (Cardoso et al., 2014; Zhang et al., 2014), and tomato (Zhang et al., 2018). Interestingly, whereas most dicotyledonous plant species hold a single copy of *MAX1*, grass genomes usually contain several copies suggesting more specialized functions of the corresponding enzymes (Challis et al., 2013).

First identified as stimulating the seed germination of root parasitic plants (Cook et al., 1966), SLs were later shown to promote the branching of arbuscular mycorrhizal fungi (AMF; Akiyama et al., 2005). Concomitantly, genetic studies highlighted the contribution of SLs in the inhibition of shoot branching (Booker et al., 2005; Gomez-Roldan et al., 2008; Umehara et al., 2008) but also as regulators of a number of other plant developmental processes such as primary root growth, secondary growth, and leaf senescence (reviewed in Al-Babili and Bouwmeester, 2015). In the last few years, the role of SLs in plant–microbe interactions beyond mycorrhization has been investigated either through *in vitro* tests to evaluate potential direct effects on microorganisms or by using biosynthetic or signaling plant mutants (reviewed in López-Ráez et al., 2017). *In vitro* studies, mostly using racemic mixtures of the synthetic analog GR24 (Johnson et al., 1981), were conducted on diverse plant–pathogenic fungal species covering both the diversity of trophic habits with host plant cells—biotrophy, hemibiotrophy, and necrotrophy—and the targeted plant part (root or shoot). If some work showed the ability of SLs to stimulate hyphal branching of *Colletotrichum acuctatum*, *Sclerotinia sclerotiorum*, *Alternaria alternata*, and *Fusarium solani* (Dor et al., 2011; Decker et al., 2017), others observed no impact of these molecules in *Rhizoctonia solani*, *Verticillium dahlia*, *Cladosporium* sp. (Steinkellner et al., 2007), or *Pythium irregular* (Blake et al., 2016). Furthermore, contradictory results were obtained for a few fungal species such as *Botrytis cinerea* (Steinkellner et al., 2007; Dor et al., 2011; Torres-vera et al., 2014; Belmondo et al., 2017) and *Fusarium oxysporum* (Steinkellner et al., 2007; Dor et al., 2011; Foo et al., 2016), leading to an unclear overall picture. *In planta* assays using comparative studies between wild-type (WT) and SL biosynthetic or signaling plant mutants, led to less contrasted results concluding, in numerous cases, on a role of SLs in disease resistance (Torres-vera et al., 2014; Piisilä et al., 2015; Stes et al., 2015; Decker et al., 2017; Xu et al., 2019). Nevertheless, as for *in vitro* assays, some work could not show any impact of SLs (Blake et al., 2016; Foo et al., 2016) and very recent work on root-knot nematodes both belonging to the *Meloidogyne* genus led to contradictory results in tomato (*Meloidogyne incognita*; Xu et al., 2019) and rice (*Meloidogine graminicola*; Lahari et al., 2019) despite the use of similar experimental approaches. In conclusion, if SL are mostly involved in plant resistance to pathogenic microorganisms, their clear role in plant–pathogens interactions is not fully depicted yet.

In a previous transcriptomic analysis, we have identified the *B. distachyon Bradi1g75310* gene as specifically induced following infection by a DON-producing *F. graminearum* strain (*Fg*DON^+^) but not by a mutant strain unable to produce the mycotoxin (*Fg*DON^–^) (Pasquet et al., 2014). Interestingly, this gene encodes a CYP similar to *A. thaliana* MAX1 previously demonstrated to be necessary to metabolize CL into carlactonoate (Abe et al., 2014). Our results strongly suggest that the *Bradi1g75310* gene encoding BdCYP711A29 is involved in orobanchol biosynthesis and that its overexpression increases FHB susceptibility in *B. distachyon*.

## Materials and Methods

### Plant Material and Growth Conditions

The *B. distachyon* WT ecotype Bd21-3 (Vogel et al., 2009) and all *B. distachyon* lines generated or selected in this study (Table 1) were cultivated as described in Pasquet et al. (2016). For expression studies, roots and leaves were collected from three week-old plants whereas spikes were collected from 5 week-old plants (mid-anthesis stage plus 96 h that is the same stage as the one chosen for expression analysis of defense genes). For hydroponic cultures, the palea and lemma of each seed were removed and seeds were surface sterilized by incubation in a 0.6% sodium hypochlorite solution for 5 min with gentle shaking followed by three rinses in sterile distilled water. Sterilized seeds were subsequently incubated for 5 days at 4°C in sterile distilled water then pre-germinated on filter paper soaked with sterile water for three days at 24°C, both steps in the dark. Seedlings were transferred on hydroponic boxes (Araponics system, http://www.araponics.com/) in sterile self-made liquid $\frac{1}{4}$ Murashige and Skoog medium [2.1 mM NH_4_NO_3_, 1.9 mM KNO_3_, 0.3 mM CaCl_2_.2H_2_O, 0.15 mM MgSO_4_.7H_2_O, 10 μM H_3_BO_4_, 10 μM MnSO_4_.H_2_O, 0.5 μM KI, 3 μM ZnSO_4_.7H_2_O, 0.1 μM Na_2_MoO_4_.2H_2_O, 0.01 μM CoCl_2_.6H_2_O, 0.01 μM CuSO_4_.H_2_O, 10 μM FeCl_2,_ 1 mM KH_2_PO_4_, (Murashige and Skoog, 1962)] for 4 weeks, with the medium changed every 3 days. One week before recovery of exudates for SL detection and quantification assays, plants were transferred to sterile self-made phosphate deficient (-KH_2_PO_4_) $\frac{1}{4}$ MS.

**TABLE 1:** List and characteristics of the *B. distachyon* lines used in this study.

**Line**	**Features**	**References**
Bd21-3	Wild-type (WT) ecotype	Vogel et al., 2009
OE-CYP11.29	Line overexpressing the *BdCYP711A29* gene	This study
OE-CYP12.20	Line overexpressing the *BdCYP711A29* gene	This study
NS-11.26	Null-segregant line recovered during the same *in vitro* culturing steps as the overexpressing lines	This study
M5374#135	TILLING mutant carrying the C1888T transition in the *BdCYP711A29* leading to a STOP codon (R450*)	This study
WT5374#139	Control line of the M5374#13 TILLING mutant carrying a wild-type allele of the *BdCYP711A29* gene	This study
M8687#12	TILLING mutant carrying the C1831T transition in the *BdCYP711A29* leading to a P431S substitution	This study
WT8687#2	Control line of the M8687#12 TILLING mutant carrying a wild-type allele of the *BdCYP711A29* gene	This study

### Binary Vector Construction and *Brachypodium distachyon* Transformation

The *BdCYP711A29* complementary DNA (cDNA) was amplified from spikelet cDNAs using primers BdCYP711A29_BI5’ATG and BdCYP711A29_EV3’TAA (Supplementary Table 1), adding a *Bam*HI restriction site at the 5’-end and an *Eco*RV restriction site at the 3’-end, respectively. The PCR product was digested using the *Bam*HI and an *Eco*RV restriction enzymes, purified using a NucleoSpin^®^ Gel and PCR Clean-up kit (Macherey-Nagel EURL, Hoerdt, France) using the manufacturer’s instructions then ligated into the pENTR1A plasmid linearized by the same restriction enzymes. The resulting pEntry-CYP711A29 plasmid was used to transfer the *BdCYP711A29* cDNA fragment into the pIPKb002 binary vector (Himmelbach et al., 2007) by *in vitro* recombination using the Gateway^®^ LR Clonase^®^ II Enzyme mix according the manufacturer’s recommendations (Invitrogen^TM^, Life Technologies SAS, Saint-Aubin, France). The resulting construct, named pIPKb002::BdCYP711A29, carried both the *BdCYP711A29* full-length cDNA under the control of the *Zea mays* ubiquitin promoter (ZmUbi) and a hygromycin resistance expression cassette allowing selection of the primary transformants.

The pIPKb002::BdCYP711A29 binary vector was then electroporated into *Agrobacterium tumefaciens* (AGL1 strain). The Bd21-3 WT line was genetically transformed using a method adapted from that described by Vogel and Hill (2008) and Alves et al. (2009). Selection of the transformants and segregation analysis were conducted as described in Pasquet et al. (2016).

### Screening of TILLING Mutants Collection

Two thousand five hundred mutant families the *B. distachyon* TILLING mutant collection^1^ available at the Institute of Plant Sciences Paris Saclay (Orsay, France) were screened for point mutations *via* Illumina^®^ sequencing of a 401-bp fragment located at the 3′ end of the *BdCYP711A29* gene encompassing a region encoding important domain of the C-terminal part of the protein: the P(E)R(F) signature, the EER triad and the heme binding domain (Supplementary Figures 1, 2). Primers used for the generation of the *BdCYP711A29* PCR product were CYP711A29-F1 and CYP711A29-R (Supplementary Table 1).

### *Fusarium graminearum* Strains, Maintenance and Spore Production

*Fusarium graminearum* strain PH-1 (*Fg*DON^+^) and the *Δtri5* mutant strain MU102 (*Fg*DON^–^; Cuzick et al., 2008) unable to produce DON were cultured as described in Pasquet et al. (2016).

### *In vitro* Orobanchol or Exudates Assay on *F. graminearum* Macroconidia

An average of 1,000 *F. graminearum* macroconidia were plated on water agar slides containing either 0.01% DMSO or various concentrations of (*rac*)-orobanchol (OlChemIm, Olomouk, Czech Republic) diluted into 0.01% DMSO. Slides were further incubated at 26°C in a moist environment for 12 h. Germ tubes were then counted on a minimum of 500 macroconidia per condition per replicate through observation on an Axio Zoom V.16 under bright field light (Zeiss, Marly-le-Roi, France). To determine the impact of exudates of different *B. distachyon* lines on *F. graminearum* macroconidial germination, macroconidia at a final concentration of 10^5^ macroconidia/mL were directly incubated in tubes containing 2 mL of each recovered exudate. After incubation for 12 h at room temperature, the germination percentage was determined on a minimum number of 1,000 macroconidia. For each experiment, three biological replicates were performed.

### Spike Treatments

For DON application, a single floret per spike at mid-anthesis was inoculated by a mixture containing 2 μg of the myxotoxin (Sigma-Aldrich, Saint-Quentin Fallavier, France) in 5 μL of a mixture of acetonitrile (1.65 μL) and 0.01% Tween 20 (1.35 μL). The control condition corresponded to spikes inoculated with the same mixture without the mycotoxin. For fungal infection, whole spikes at mid-anthesis were sprayed with the fungal spore suspension (1 × 10^5^ conidia/mL), until dripping. Inoculated plants were covered with clear plastic bags for which the internal face had been sprayed with distilled water beforehand. The first 24 h inoculated heads were kept in the dark, then incubated with a photoperiod of 16 h light and 8 h darkness at 20°C with the same light intensities as those used for plant development (Pasquet et al., 2016). Applications of 0.01% Tween 20 was performed as control condition for each inoculation experiment. Symptoms were observed at 7 and 14 days after spraying of the conidial suspension. A spikelet was considered as symptomatic if at least half of its florets were symptomatic.

### RNA Extraction

Leaves from three 2-week old plants plant or five spikelets from independent plants were ground in liquid nitrogen and total RNA was extracted from 0.1 g of the resulting powder using TRIzol^®^ (Invitrogen, Life Technologies SAS, Saint-Aubin, France) followed by an RNase-free DNase I step (Ambion^®^, Applied Biosystems, Courtaboeuf, France) according to manufacturers’ instructions. Total RNA was further purified using the NucleoSpin RNA Clean-up XS kit (Macherey-Nagel, Hoerdt, France).

### Fungal DNA Quantification by qPCR

For quantification of *F. graminearum* DNA, 10 spikes spray-inoculated with either of the two strains used in this study were pooled per time-point. Genomic DNA was extracted as described in Pasquet et al. (2016). Quantification of fungal DNA was realized by qPCR (see below) on 10 ng of total DNA using primers specific for the 18S ribosomal subunit-encoding genomic region (Mudge et al. (2006; Supplementary Table 2).

### Real-Time PCR

Complementary DNA synthesis was performed on 1 μg of total RNA using the ImProm-II^TM^ reverse transcription system (Promega France, Melun-les-Charbonnières, France) according to the manufacturer’s instructions. The resulting product was diluted 10 times in nuclease-free water. Primers were designed to amplify plant gene transcripts (Supplementary Table 2), including reference genes *Bradi4g00660* (*UBC18*) and *Bradi4g41850* (*ACT3*-like under accession number XM_003578821 in the nucleotide NCBI database) as previously determined by Hong et al. (2008); (Supplementary Table 2). qPCR reactions were performed on 2 μL of the diluted cDNA product using 8 pmoles of each specific primer and 10 μL of SYBRGreen Master Mix in a final volume of 20 μL. Reactions were performed in a Light Cycler LC480 Real-time PCR system (Roche Diagnostics, Meylan, France). All qPCR reactions were carried out on biological triplicates, each in technical duplicate. The final threshold cycle (Ct) values were the mean of three values (biological triplicates), each corresponding to the mean of technical duplicates. The comparative ΔΔCt method was used to evaluate the relative quantities of each amplified product in the samples. The Ct was automatically determined for each reaction by the Light Cycler LC480 Real-time PCR system set with default parameters. The specificity of the qPCR reactions was determined by melt curve analysis of the amplified products using the standard method installed in the system.

### Strigolactones Detection and Quantification

Detection and quantification of SLs exuded from the roots has been performed according to a method adapted from Ravazzolo et al. (2019). *B. distachyon* lines were grown hydroponically and exudation was performed in fresh $\frac{1}{4}$ MS-KH_2_PO_4_ medium during 24 h. When necessary, exudates were immediately frozen in liquid nitrogen and conserved at −80°C before analysis. SLs were extracted from liquid medium complemented with 10 ng of (*rac*)-GR24 as internal standard, by adding the same volume of ethyl acetate followed by a manual and vigorous mixing during 10 min. Organic phase was decanted and dried using a rotary evaporator (Rotavapor, Buchi). The solid phase was resuspended in acetonitrile, and conserved at −20°C before analysis according to Boutet-Mercey et al. (2017). Separation was performed on a BEHC_18_ column (2.1 × 100 mm, particle size 1.7 μm, Waters), using an ACQUITY UPLC I-class system (Waters), and detection on Waters Xevo TQ-S equipped with an ESI source and operated in positive ion mode. Identification and quantification were performed by the very sensitive MRM mode in LC-MS/MS (Supplementary Table 3), however, the signal in the sample was not sufficient to obtain a fullscan MS/MS spectrum. We first optimized all the conditions of MRM acquisition on the orobanchol standard in full scan methods (Supplementary Figure 3A), and chose the two main MRM transitions, then we analyzed the sample with this MRM methods on both transitions to guarantee the identification (Supplementary Figure 3B).

### Phylogenetic Analyses

Amino acid sequences were recovered from The Cytochrome P450 Homepage (Nelson, 2009) or through reciprocal BLAST analysis for the *Hordeum vulgare* protein sequences. Phylogenetic analyses were inferred from a multiple sequence alignment generated by CLUSTAL W and by using the Maximum Likelihood method based on the JTT matrix-based model, conducted on MEGAX software (Kumar et al., 2018).

## Results

### The *Bradi1g75310* Gene Belongs to an Oligogenic Family Homologous to the Arabidopsis *MAX1* Gene and Is Specifically Induced Under *F. graminearum* Infection in a DON-Dependent Manner

**FIGURE 1:**
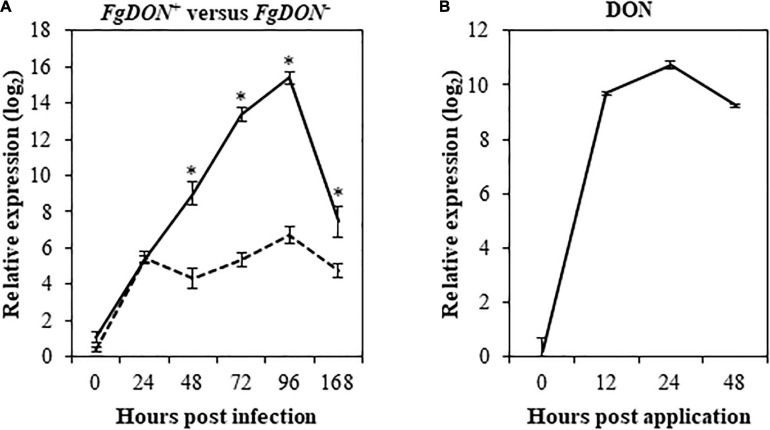
The *Bradi1g75310* (*BdCYP711A29*) gene is transcriptionally induced during FHB and following DON treatment. Relative quantification of *Bradi1g75310* transcripts in the Bd21-3 (WT) ecotype of *B. distachyon* following *F. graminearum* infections or following DON treatment. **(A)** *Bradi1g75310* expression level (fold-change. log_2_) following point infection with the *FgDON*^+^ (solid line) or with the *FgDON*^–^ (dashed line) strain of *F. graminearum* compared to mock treatment. **(B)** *Bradi1g75310* expression level (fold-change. log_2_) following DON treatment compared to mock treatment. The relative quantity of the *Bradi1g75310* transcripts compared to mock condition was calculated using the comparative cycle threshold (Ct) method (2^–ΔΔ*Ct*^). The *B. distachyon UBC18* and *ACT7* genes (*Bradi4g00660* and *Bradi4g41850*) were used as endogenous controls to normalize the data for differences in input RNA between the different samples. Mean of three independent biological replicates ± standard deviation. Asterisks indicate significant differences between conditions (Student’s *t*-test *p* value < 0.05).

A previous study has identified *Bradi1g75310* as a gene induced by a DON-producing *F. graminearum* [*Fg*DON^+^, 6.93 fold log_2_ compared to the mock condition 96 hours post inoculation (hpi)] while it does not respond to a mutant strain unable to produce the mycotoxin [*Fg*DON^–^, 0.77 fold log_2_ compared to the mock condition (96 hpi); Pasquet et al., 2014]. In order to validate these transcriptomic data, reverse transcription-quantitative PCR (RT-qPCR) was conducted on the same RNA samples as those used for microarrays. Results showed good correlation between microarrays and RT-qPCR data as the *Bradi1g75310* gene was strongly induced by the *Fg*DON^+^ strain (15.4 fold log_2_ at 96 hpi) and far less (6.2 fold log_2_ at 96 hpi) by the *Fg*DON^–^ strain (Figure 1A). To determine whether expression could also be induced by the mycotoxin itself, similar experiments were conducted on spikes point-inoculated with DON along kinetics from 0 to 48 hours post application (hpa). Expression of the *Bradi1g75310* gene was strongly and rapidly induced by DON reaching a maximum of 10.74 induction fold log_2_ 24 hpa (Figure 1B). Overall, these results confirm that the *Bradi1g75310* gene expression is strongly induced by DON either produced by *F. graminearum* during infection or as a commercial molecule. The rapid and strong induction of expression following application of the mycotoxin alone reinforces the idea of a direct inductive action of the molecule.

**FIGURE 2:**
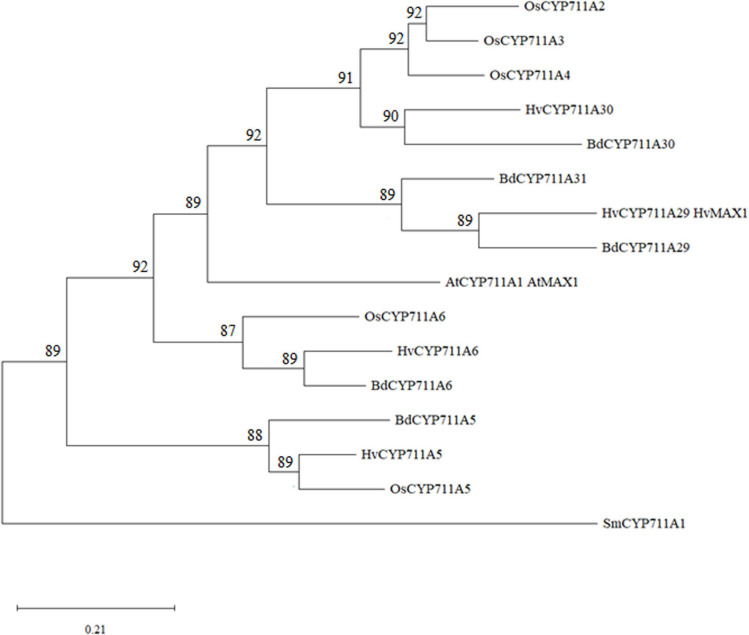
Molecular phylogenetic analysis of *A. thaliana*, *B. distachyon, H. vulgare*, *O. sativa*, and *S. moellendorffii* CYP711As. The protein evolutionary history was inferred by using the Maximum Likelihood method based on the JTT matrix-based model (Jones et al., 1992). The bootstrap consensus tree inferred from 500 replicates (Felsenstein, 1985) is taken to represent the evolutionary history of the taxa analyzed (Felsenstein, 1985). Branches corresponding to partitions reproduced in less than 50% bootstrap replicates are collapsed. The percentage of replicate trees in which the associated taxa clustered together in the bootstrap test (500 replicates) are shown next to the branches (Felsenstein, 1985). Initial tree(s) for the heuristic search were obtained automatically by applying Neighbor-Join and BioNJ algorithms to a matrix of pairwise distances estimated using the JTT model, and then selecting the topology with superior log likelihood value. This analysis involved 16 amino acid sequences. There were a total of 567 positions in the final dataset. Evolutionary analyses were conducted in MEGA X (Kumar et al., 2018). The tree has been rooted with *Selaginella moellendorffii* CYP711A1 (SmCYP711A1). At: *A. thaliana*; Bd: *B. distachyon*; Hv: *Hordeum vulgare*; Os: *O. sativa;* Sm: *S. moellendorffii*. Protein sequences used in this analysis are available under the following accession numbers: AtCYP711A1, OAP07831.1; BdCYP711A5, XP_003571126.1; BdCYP711A6: XP_003560652.1; BdCYP711A29, XP_003562092.2; BdCYP711A30: XP_003575594.2; BdCYP711A31, XP_010237353.2; HvCYP711A5, BAJ97619.1; HvCYP711A6, KAE87888859.1; HVCYP711A29, BAJ98237.1; HvCYP711A30, KAE8810993.1; OsCYP711A2, XP_015633367.1; OsCYP711A3, XP_015644699.2; OsCYP711A4, XP_015642272.1; OsCYP711A5, XP_015626073.1; OsCYP711A6, XP_015644019.1; SmCYP711A1, XP_002972055.1.

The *Bradi1g75310* gene is 2178 base pairs (bp) long, including 48 and 18 bp of 5′- and 3′-untranslated regions (UTR), respectively. The nucleotide sequence is constituted of 5 exons and 4 introns and the 1,572-bp coding sequence encodes a putative 523 amino acid CYP (Supplementary Figure 1), which has been classified as belonging to the CYP711A subfamily and named BdCYP711A29 (Nelson, 2009).^2^ This notation will be therefore used throughout this work to refer both to the protein (BdCYP711A29) and the gene (*BdCYP711A29*). According to this database, BdCYP711A29 belongs to an oligomeric subfamily comprising 4 other members: BdCYP711A5 (*Bradi3g08360*), BdCYP711A6 (*Bradi1g37730*), BdCYP711A30 (*Bradi4g08970*), and BdCYP711A31 (*Bradi4g09040*). Sequences from *A. thaliana* and *Oryza sativa* CYP711A proteins (1 and 5 members, respectively) were used together with *B. distachyon* CYP711A protein sequences to perform multiple protein sequence alignment of the full-length proteins and phylogenetic analysis. The CYP711A1 sequence from the non-vascular plant species *Selaginella moellendorffii* has been used to root the tree (Figure 2 and Supplementary Table 4). BdCYP711A5, HvCYP711A5 and *O. sativa* OsCYP711A5, as well as BdCYP711A6, HvCYP711A6, and OsCYP711A6, constitute two specific clades. The other *B. distachyon*, *H. vulgare*, and *O. sativa* CYP711A proteins [BdCYP711A29, BdCYP711A30, BdCYP711A31, HvCYP711A29 (also named HvMAX1), HvCYP711A30, OsCYP711A2, OsCYP711A3, and OsCYP711A4] strongly grouped with *A. thaliana* MAX1 (*At2g26510*), and were distributed in two subclades. One subclade contains all rice proteins and the barley and *B. distachyon* CYP711A30 proteins and exhibits a strong species structuration. The BdCYP711A29 protein belongs to the other subclade together with the barley HvCYP711A29 and the BdCYP711A31 protein. The BdCYP711A29 protein exhibits 55.8% with the *A. thaliana* MAX1 protein involved in SL biosynthesis, converting CL into carlactonoic acid (CLA; Abe et al., 2014). BdCYP711A29 also shares between 46 and 58% identity with *O. sativa* CYP711A proteins (A2, A3, A4), all but one recently demonstrated to be involved at different steps of SL biosynthesis downstream of CL (Cardoso et al., 2014; Zhang et al., 2014).

**FIGURE 3:**
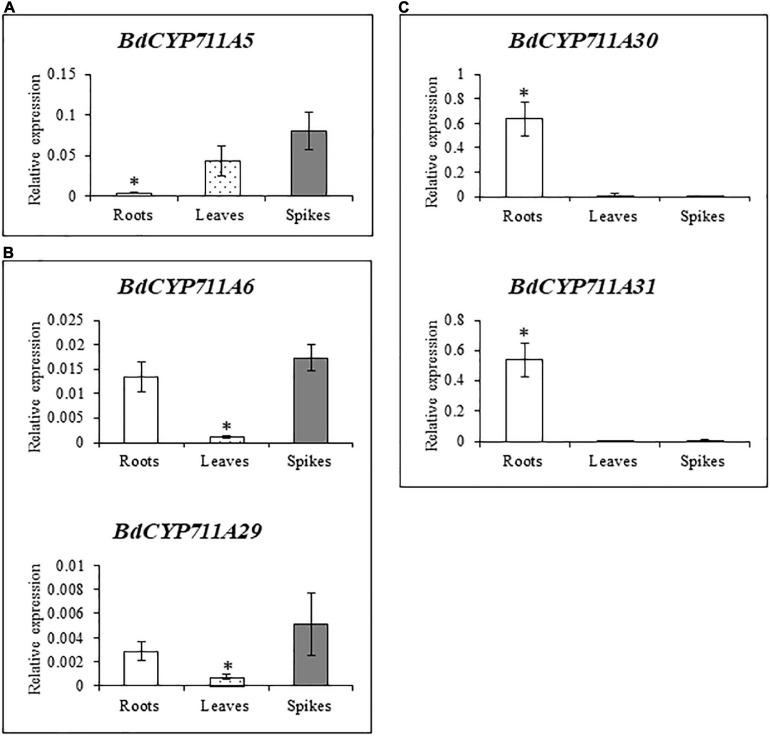
Expression pattern of the 5 *BdCYP711A* genes in roots, leaves, and spikes. **(A)**, **(B)**, and **(C)** correspond to different expression patterns, mentioned as categories in the main text. Relative quantification of transcripts of the *BdCYP711A5* (*Bradi3g08360*), *BdCYP711A6* (*Bradi1g37730*), *BdCYP711A29* (*Bradi1g75310*), *BdCYP711A30* (*Bradi4g08970*), and *BdCYP711A31* (*Bradi4g09040*) genes in different organs of the Bd21-3 (WT) ecotype of *B. distachyon*: roots and leaves were collected from 3 week-old plants under hydroponic conditions in ½ MS liquid medium and spikes were collected at mid-anthesis on plants grown in pots under standard conditions. The relative quantity of transcripts was calculated using the comparative cycle threshold (Ct) method (2^– Δ*Ct*^) using the *B. distachyon UBC18* and *ACT7* genes (*Bradi4g00660* and *Bradi4g41850*) as endogenous controls to normalize the data for differences in input RNA between the different samples. Mean of three independent biological replicates ± standard deviation. For each gene, asterisks indicate significant differences between organs (*p value < 0.05, Student’s *t*-test).

In order to better characterize the *B. distachyon CYP711A* gene family, the relative expression of the five genes was examined by RT-qPCR in three week-old roots and leaves, and spikes of five week-old plants. Three different categories could be depicted based on the CYP711A gene expression pattern (Figure 3). The first one, with *BdCYP711A5* gene as a single member, exhibits an overall low expression level with stronger expression in leaves and spikes (Figure 3A). *BdCYP711A6* and *BdCYP711A29* genes belong to the second category characterized similarly to the first one by a low expression level, and a significantly higher expression in roots and spikes (Figure 3B). The third category contains the *BdCYP711A30* and *BdCYP711A31* genes showing higher expression in roots only (Figure 3C). These results therefore indicate that the five *BdCYP711A* genes exhibit different spatial expression in the plant.

As mentioned before, previous transcriptomic data indicated that *BdCYP711A29* is the only member of this oligogenic family to be differentially expressed following *F. graminearum* infection in a DON-dependent manner. To validate these data, RT-qPCR experiments using primers specific of the *BdCYP711A5*, *BdCYP711A6*, *BdCYP711A30*, and *BdCYP711A31* genes (Supplementary Table 2) were conducted on the same samples as for the *BdCYP711A29* gene. Following DON application, no significant induction of expression was detected for any of the four genes (Supplementary Figure 4A) apart from a decreased expression of the *BdCYP711A6* gene from 12 hpa compared with the mock-inoculated control spikes. The expression of the *BdCYP711A5*, *BdCYP711A30*, and *BdCYP711A31* genes was induced following infection by the *F. graminearum FgDON+* strain, at different timepoints: [from 168 hpi onward for *BdCYP711A5*, 48 hpi for *BdCYP711A30* and 96 hpi for *BdCYP711A31* (Supplementary Figure 4B)]. However, besides the difference in its timing, the magnitude of induction is by no means equivalent to the one observed for the *BdCYP711A29* gene (Figure 1). Indeed, whereas the maximal level of induction of *BdCYP711A29* at 48 hpi reaches more than 15-fold log2 (that is more than 18,000 fold in absolute values, Figure 1A), maximal levels of expression for the *BdCYP711A5*, *BdCYP711A30*, and *BdCYP711A31* correspond at most to a 5, 6.5, and 17-fold induction compared with the mock-inoculated control (Supplementary Figure 4B). From these results, we can conclude that, among the five *BdCYP711A* genes, *BdCYP711A29* is the only gene copy which exhibits such a strong transcriptional induction following *F. graminearum* infection in a DON-dependent manner.

### Construction of *B. distachyon* Lines Overexpressing *BdCYP711A29* and Selection of *BdCYP711A29* TILLING Mutants

**FIGURE 4:**
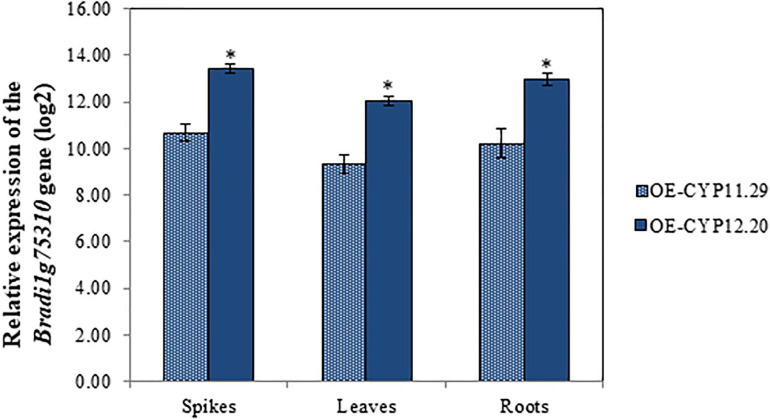
Molecular characterization of *B. distachyon* lines overexpressing the *BdCYP711A29* gene. Relative expression of the *BdCYP711A29* gene in spikes. leaves and roots of overexpressing lines OE-CYP11.29 and OE-CYP12.20. The relative quantity of *BdCYP711A29* transcripts (fold-change. log_2_) of OE lines as compared with the Bd21-3 WT line was calculated using the comparative cycle threshold method (2^– ΔΔ*Ct*^). The *B. distachyon UBC18* and *ACT7* genes (*Bradi4g00660* and *Bradi4g41850*) were used as endogenous controls to normalize the data for differences in input RNA between the different samples. Data represent mean values of three independent biological experiments (*n* = 3) and two technical repetitions ± standard deviation. Different letters indicate significant differences between conditions (Student’s *t*-test *p* value < 0.01).

Following *Agrobacterium tumefaciens*-mediated transformation of the *B. distachyon* Bd21-3 ecotype (see section “Materials and Methods”), two independent homozygous *BdCYP711A29* overexpressing lines were recovered (data not shown),. In addition, a null segregant, NS-11.26, that is a plant regenerated from the *in vitro* culture steps but devoid of the transgene, was selected to be used as a control (Table 1). RT-qPCR experiments were conducted to verify the expected overexpression of the *BdCYP711A29* gene in spikes, leaves and roots. As shown in Figure 4, overexpression rates were highly similar in the three organs with line OE-CYP12.20 exhibiting a significantly higher level of expression than line OE-CYP11.29. These two overexpressing lines were used in further experiments (Table 1).

Lines mutated for the *BdCYP711A29* gene were obtained by screening the *B. distachyon* TILLING mutant collection available in the Bd21-3 ecotype (Dalmais et al., 2013, see section “Materials and Methods” and Supplementary Figures 1, 2). Eleven mutant families carrying point mutations in the region of interest were recovered (Supplementary Table 5). A Sorting Intolerant From Tolerant (SIFT) analysis (Kumar et al., 2009) showed that four mutant families exhibited a SIFT score below 0.05 and were therefore likely to carry point mutations predicted to strongly impact the functionality of the protein (Supplementary Table 5): three families exhibited missense mutations (8,687, 7,708, and 7,424) and one (5,374) carried a nonsense mutation (R450*) leading to a protein truncated of the last 73 amino-acids, therefore lacking the highly conserved heme-binding domain (Supplementary Figure 2). Control lines carrying a *BdCYP711A29* WT allele could be selected for families 8,687 and 5,374 but not for two other mutant families 7,424 and 7,708, which is problematic considering the numerous mutations per genome in TILLING mutant families. Therefore, only the two mutant lines, notated M8687#12 and M5374#135, respectively, and their corresponding controls, notated WT8687#2 and WT5374#139, respectively, were considered for further analyses (Table 1).

### *BdCYP711A29* Overexpression Increases Orobanchol Exudation From *B. distachyon* Roots

As previously mentioned BdCYP711A29 belongs to the CYP711A subfamily and is closely related to *A. thaliana* MAX1 and rice MAX1-like CYPs (Figure 2). Members of this subfamily are involved in SL biosynthesis (Bak et al., 2011; Stirnberg et al., 2002; Booker et al., 2005; Challis et al., 2013; Zhang et al., 2014). However, as for other multicopy families of proteins involved in plant secondary metabolism, phylogeny is usually insufficient to demonstrate functional homology (Moghe and Kruse, 2018). To determine whether the *B. distachyon* BdCYP711A29 is involved in SL biosynthesis, SL quantification was performed from root exudates.

First, we performed preliminary experiments on root exudates from the WT line collected after 1 week of phosphate starvation using ethyl acetate supplemented with 10 ng (*rac*)-GR24 as the internal standard (see section “Materials and Methods” and Supplementary Figure 3). Analyses were performed by LC-MS/MS *via* Multiple Reaction Monitoring (MRM). Supplementary Table 3 lists the chromatographic peaks of characteristic *m/z* MRM transitions detected at least in one biological replicate as compared to blank sample. Both orobanchol- and strigol-type SLs were found but most of detected transitions were not specific to one SL (e.g., 345 > 97, 345 > 248 and 367 > 270 could be linked to both didehydroorobanchol and didehydrostrigol, Supplementary Table 3). We therefore focused on compounds detected through at least two different characteristic transitions: orobanchol, orobanchyl acetate, solanacol, and solanacyl acetate, each giving a chromatographic peak at only one retention time (RT). Among these, only orobanchol characteristic transitions were recorded at the same RT (9.2 min). An additional experiment using the WT line allowed us to detect a chromatographic peak at the same RT (9.2 min) for 3 orobanchol characteristic transitions (347 > 205; 347 > 97; and 347 > 233). Finally, this RT was confirmed by adding orobanchol standard in samples, which resulted in a single peak at this same RT for each transition and similar ratios as in WT line (Supplementary Figure 3B). Therefore, following MRM, we were able to confirm the presence of orobanchol in *B. distachyon* WT root exudates.

**FIGURE 5:**
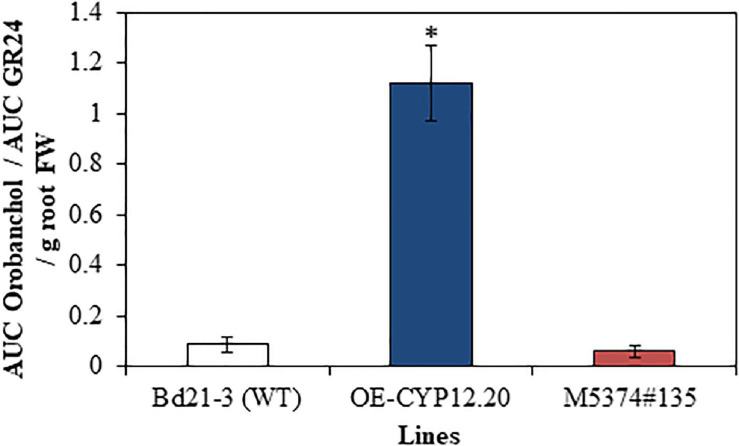
Quantification of orobanchol in *B. distachyon* exudates. Relative quantification of orobanchol in root exudates of WT (Bd21-3), overexpressing (OE-CYP12.20), and mutant (M5374#135) lines after 7 days of phosphorus starvation. AUC, Area under the curve. MRM transitions 321 > 224 and 347 > 97 were used to quantify GR24 and orobanchol signals, respectively. Mean of three biological replicates ± standard deviation. The asterisk indicates significant differences between conditions (*p*-value < 0.05, Tukey’s test).

Second, the relative amount of orobanchol exuded from the roots of three selected contrasting lines, the WT, overexpressing (OE-CYP12.20) and TILLING mutant (M5374#135) lines was then measured by calculating the ratio between the area under the curve (AUC) of the 347 > 97 chromatographic peak, specific to orobanchol, and the AUC of the 321 > 224 chromatographic peak, and comparing it to values of the internal standard (*rac*)-GR24 (Boutet-Mercey et al., 2017). Data were normalized over root fresh weight. We observed a significant increase of about 16 times of the relative quantity of orobanchol in OE-CYP12.20 exudates, as compared to WT and M5374#135 lines (Figure 5 and Supplementary Table 6). These results strongly suggest that the *B. distachyon* BdCYP711A29 protein is involved in orobanchol biosynthesis. No significant difference was observed between Bd21-3 and the TILLING mutant line, suggesting potential functional redundancy with one or several *B. distachyon* other CYP711A copies.

### Overexpression of the *BcCYP711A29* Gene Increases Susceptibility to FHB but Has no Impact on Plant Development

Considering that the *B. distachyon* BdCYP711A29 is involved in orobanchol biosynthesis, 4 weeks-old (end of the vegetative stage in our conditions) plants of overexpressing lines and TILLING mutants and their respective control lines were examined for the number of tillers and compared to the WT Bd21-3 ecotype. Apart from control and mutant lines WT5374#139 and M5374#135, no statistically significant difference could be observed compared to the WT line (Supplementary Figure 5). This indicates that BdCYP711A29 does not significantly participate to *B.distachyon* development.

**FIGURE 6:**
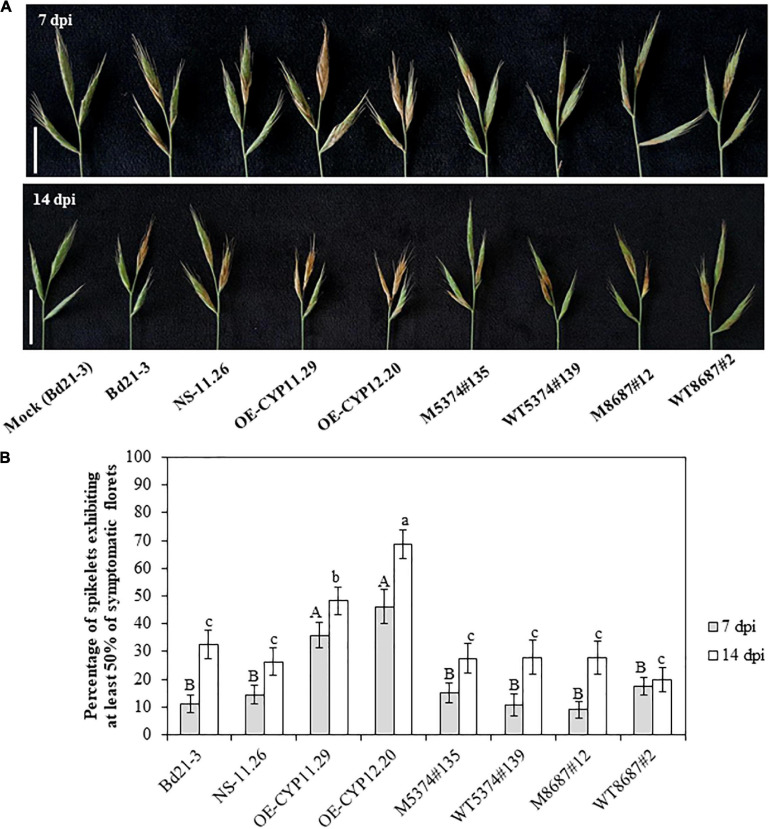
Overexpression of the *BdCYP711A29* gene increases FHB susceptibility following spray inoculation of *F. graminearum*. **(A)** Typical FHB symptoms at 7 and 14 days following spray inoculation of whole spikes of the different lines by the *F. graminearum FgDON*^+^ strain. Bars equal 1 cm. **(B)** Percentage of spikelets exhibiting FHB symptoms on at least 50% of the florets of the inoculated spikes at 7 and 14 dpi by the PH-1 strain. Mean of four independent biological replicates (*n* = 52). Different letters indicate significant differences between conditions; upper case and lower case letters indicate the statistical comparison at 7 and 14 dpi, respectively (*p*-value < 0.05, Tukey’s test).

In order to determine whether the *BdCYP711A29* gene is involved in the interaction between *B. distachyon* and *F. graminearum*, spray inoculations by spore suspensions of the *F. graminearum Fg*DON^+^ strain were performed on whole spikes of the lines previously described: WT, overexpressing lines (OE-CYP11.29 and OE-CYP12.20) and the corresponding null segregant (NS-11.26), TILLING mutant lines (M5374#135, M8687#12) and their control lines (WT5374#139 and WT8687#2, respectively). Representative symptoms, that is more or less extended bleaching on spikelets, at 7 and 14 dpi are presented in Figure 6A. FHB symptoms were quantified at 7 and 14 dpi by counting the number of spikelets exhibiting more than 50% of symptomatic florets over the total number of spikelets (Figure 6B). The results represent the mean of four independent biological replicates. Seven dpi, both overexpressing lines OE-CYP11.29 and OE-CYP12.20 exhibited more developed symptoms (35.9 ± 4.7% and 46.2 ± 6.1% of symptomatic spikelets, respectively) as compared to WT (11.2 ± 3.3%) and NS-11.26 (14.4 ± 3.3%) (Figure 6B). All the TILLING mutant lines, carrying either the mutant or WT allele for the *BdCYP71A29* gene, presented symptoms equivalent to those observed with the WT line (Figure 6B). After another week of disease development (14 dpi), the tendency described above was conserved. Both overexpressing lines presented increased symptoms as compared with control lines (48.3 ± 4.9% for OE-CYP11.29 and 68.6 ± 5.2% for OE-CYP12.20 compared to 32.5 ± 5.1% and 26.3 ± 5.0% for Bd21-3 and NS-11.26, respectively) (Figure 6B). The approx. 20% differential development of symptoms between the two overexpressing lines was statistically significant (Figure 6B). As observed at 7 dpi, the TILLING lines did not show any significant difference in the level of symptomatic spikelets as compared to the WT line (Figure 6B).

**FIGURE 7:**
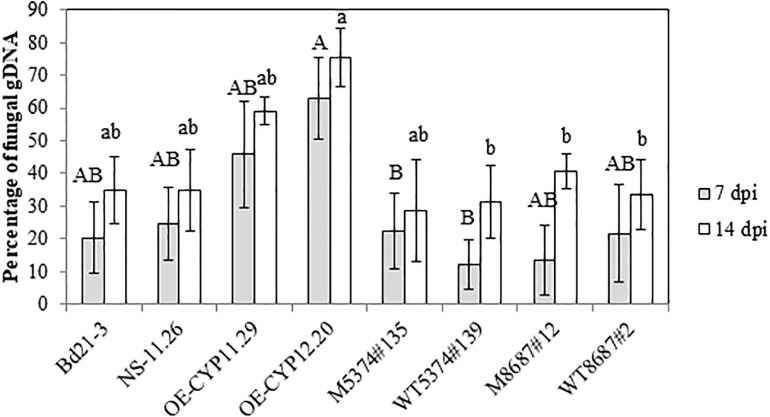
Overexpression of the *BdCYP711A29* gene promotes *F. graminearum* development on *B. distachyon* spikes following spray inoculation. Relative quantification of fungal DNA by qPCR compared to total DNA 7 and 14 days after spray inoculation of different *B. distachyon* lines with the *FgDON*^+^ strain of *F. graminearum*. Mean of three independent biological replicates ± standard error. Different letters indicate significant differences between conditions (*p*-value < 0.05, one way ANOVA and pairwise *t*-tests with Bonferroni correction).

To be able to correlate the differences of disease symptoms described above with a differential fungal development, we quantified by qPCR the relative amount of fungal gDNA on the same material used for symptoms quantification (3 out of 4 biological replicates). At 7 dpi, WT samples exhibited 20.3 ± 10.8% of fungal DNA and the other control lines showed similar values: 24.7 ± 11.3; 11.9 ± 7.6 and 21.6 ± 14.9% for NS-11.26, WT5374#139 and WT8687#2, respectively (Figure 7). Whereas TILLING mutant lines did not exhibit differences greater than 10% compared to WT and TILLING control lines (22.5 ± 11.6 and 13.4 ± 10.9% for M5374#135 and M8687#12, respectively), both overexpressing lines contained an increased percentage of fungal DNA as compared to Bd21-3 and NS-11.26 (45.8 ± 16.4 and 62.8 ± 12.5% for OE-CYP11.29 and OE-CYP12.20, respectively, Figure 7). This tendency was conserved at the 14 dpi time-point since all the control lines and TILLING mutant lines ranged from 28.7 ± 15.6 (M5374#135) to 40.6 ± 5.4% (M8687#12), contrary to overexpressing lines which exhibited from around 20–45% more fungal DNA (59.0 ± 4.3 and 75.5 ± 8.9% for OE-CYP11.29 and OE-CYP12.20, respectively, Figure 7). Statistical analyses were conducted on these results and showed that the OE-CYP lines contained significantly more fungal DNA compared to the control lines Bd21-3 and NS-11.26 either at one (OE-CYP11.29, 7 dpi) or both time-points (OE-CYP12.20, 7 and 14 dpi) therefore confirming the increased susceptibility of these lines to fungal infection (Figure 7).

### BdCYP711A29 Does Not Participate to *B. distachyon* Defenses Following *F. graminearum* Infection

**FIGURE 8:**
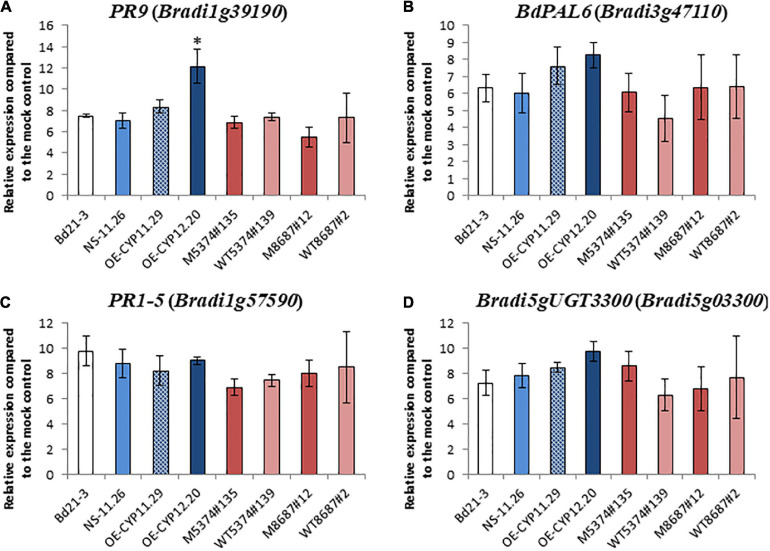
Relative defense gene expression levels in *B. distachyon* lines altered in *BdCYP711A29* following *F. graminearum* infection compared to mock condition. Relative quantification (fold-change, log_2_) of the *PR9* (*Bradi1g39190*), **(A)**, *BdPAL6* (*Bradi3g47110*), **(B)**, *PR1-5* (*Bradi1g57590*), **(C)**, and *Bradi5gUGT3300* (*Bradi5g03300*), **(D)** expression levels in the *B. distachyon* lines altered in the *BdCYP711A29* locus or gene expression 96 hours after *F. graminearum* infection (*FgDON*^+^ strain) compared to mock treatment. The relative quantity of gene transcripts compared to mock condition was calculated using the comparative cycle threshold (Ct) method (2^–ΔΔ*Ct*^). The *B. distachyon UBC18* and *ACT7* genes (*Bradi4g00660* and *Bradi4g41850*) were used as endogenous controls to normalize the data for differences in input RNA between the different samples. Mean of three independent biological replicates ± standard deviation. The asterisk **(A)** indicates significant differences (Student’s *t*-test, *p* value < 0.05). No difference was shown to be statistically significant under more stringent conditions (Student’s *t*-test, *p* value < 0.01).

To determine if the observed increased susceptibility of the overexpressing lines may be due to a modification of plant defenses, expression of *B. distachyon* defense marker genes previously shown to be induced upon *F. graminearum* infection (Pasquet et al., 2016) was investigated by RT-qPCR on spikes of the WT line, the NS-11.26 null segregant, the two overexpressing lines (OE-CYP11.29 and OE-CYP12.20), the two TILLING mutant lines (M5374#135 and M8687#12) and their corresponding controls (WT5374#139 and WT8687#2, respectively). To do so, we used four defense marker genes, two genes encode pathogenesis-related proteins, PR1-5 (Bradi1g57590, Kakei et al., 2015; Kouzai et al., 2016) and *PR9* (*Bradi1g39190*, Pasquet et al., 2014), the third one encodes a phenylalanine ammonia lyase, recently renamed *BdPAL6* (*Bradi3g47110*, Cass et al., 2015) and the last one codes for a uridine diphosphate (UDP)-glucosyltransferase shown to be involved in the conjugation of DON into DON-3-*O*-glucose (*Bradi5g03300*, Pasquet et al., 2016). The expression of these four defense genes was first assessed on healthy spikes. Very low basal levels of expression prevented proper comparison of the expression levels in healthy plants of the different genotypes (data not shown). Nevertheless, relative expression of the four marker genes could be quantified after spray inoculation by the *F. graminearum Fg*DON^+^ strain. The 96 hpi time-point was chosen because it corresponds both to the infection time-point used in the transcriptomic analysis of *B. distachyon* response to fungal infection (Pasquet et al., 2014) and to maximal expression of most of the defense genes in our conditions (Schweiger et al., 2013; Pasquet et al., 2016). Almost no statistically significant difference could be detected between the different lines, whatever the alteration of the *BdCYP711A29* gene, overexpression or mutation (Figure 8). The unique difference was observed for the *PR9* gene (*Bradi1g39190*, Figure 8A), for which a stronger induction was observed 96 hpi in the OE-CYP12.20 line as compared to the WT Bd21-3 line (1.6 fold log_2_) and to the NS-11.26 null segregant (1.7 fold log_2_). These results suggest that the increased susceptibility of lines overexpressing the *BdCYP711A29* gene likely does not result from an alteration of plant defense responses.

### Orobanchol Promotes *F. graminearum* Macroconidial Germination *in vitro*

**FIGURE 9:**
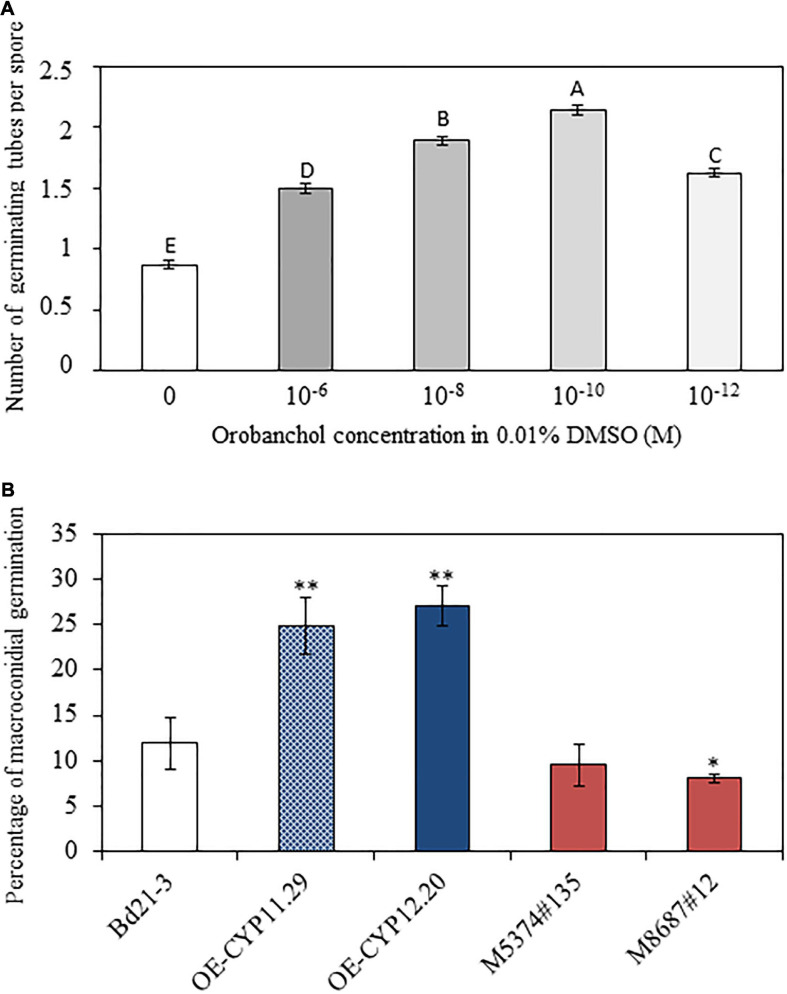
Orobanchol promotes spore germination of *F. graminearum*. **(A)** Counting of germinating tubes developed by macroconidia of the *F. graminearum FgDON^+^* strain 12h after incubation on agar medium containing different concentrations of orobanchol. 0.01% DMSO was used as a negative control as it corresponds to the dilution solution of orobanchol. Counts were performed on at least 500 macroconidia per condition and repeated in three biological replicates. Different letters indicate statistically significant differences (*p*-value < 0.01, Tukey’s test). **(B)** Percentage of macroconidial germination of the *F. graminearum FgDON^+^* strain 12 h after incubation in exudates of the different *B. distachyon* lines. Asterisks indicate significant differences compared to the wild-type line Bd21-3 (*p-value < 0.05, **p-value < 0.001, Student’s *t*-test).

Strigolactones have been shown to promote pre-symbiotic growth of AMF (Akiyama et al., 2005) and more recently to regulate root entry through the modulation of hyphopodia formation (Kobae et al., 2018). As mentioned in the Introduction part, the picture is less clear for other fungi, in particular for plant pathogenic fungi (Steinkellner et al., 2007; Dor et al., 2011; Torres-vera et al., 2014; Foo et al., 2016; Belmondo et al., 2017; Decker et al., 2017). No major difference in plant defense gene expression could explain the increased susceptibility of lines overexpressing the *BdCYP711A29* (Figure 8) and the OE-CYP12.20 line overexpressing the *BdCYP711A29* gene exudates increased levels of orobanchol (Figure 5). We therefore investigated the direct impact of this SL on *F. graminearum*, and more specifically on the germination of typical 5–6 cell asexual spores named macroconidia (Booth, 1975). Various concentrations of orobanchol were tested and their impact on the germination of *F. graminearum* macroconidia was estimated using an *in vitro* test on small agar slides. As shown in Figure 9A (see also Supplementary Figure 6 for characteristic macroconidial germination patterns at each orobanchol concentration), orobanchol increased the number of fungal germ tubes per macroconidium after 12 h incubation with a peak at 10^–10^ M. These results show that orobanchol stimulates *F. graminearum* macroconidial germination.

To further establish the relationship between alteration of the *BdCYP711A29* gene or of its expression and *F. graminearum* macroconidial germination, similar experiments were conducted using root exudates of the WT, overexpressing (OE-CYP11.29 and OE-CYP12.20) or mutant (M5374#135 and M8687#12) lines obtained in phosphate-deprived conditions (see section “Materials and Methods” and Figure 9B) in place of pure orobanchol. After 12 h incubation at room temperature, germination percentages were 11.90 ± 2.83% if exudates were obtained from Bd21-3 (WT) roots (Figure 9B). The values were significantly increased if root exudates from the OE-CYP11.29 (24.90 ± 3.20%) or the OE-CYP12.20 (27.15 ± 2.23%) lines were used (Figure 9B). No significant differences were observed with root exudates from the mutant line M5374#135 (9.51 ± 2.30) nevertheless a significant difference (reduction) observed for the mutant line M8687#12 (7.05 ± 0.43) (Figure 9B). These results show that altering the *BdCYP711A29* gene sequence or expression modifies the composition of root exudates and consequently their ability to promote *F. graminearum* macroconidial germination. Together with the data obtained with commercial orobanchol (Figure 9A) and the analysis of SL amounts shown previously (Figure 5), the modification of germination presumably occurs through a modulation of orobanchol content.

## Discussion

Fusarium Head Blight is primarily due to the ascomycete fungus *F. graminearum* and represents one of the most damaging diseases on small-grain cereals in temperate areas (Dweba et al., 2017). It induces yield losses and is a major public health issue due to the production by the fungus of mycotoxins harmful to humans and animals (Chen et al., 2019). Plant–pathogen interactions have been shown to involve phytohormones, the main ones being SA, JA, and ET (Glazebrook, 2005). Numerous studies using transcriptomics and/or metabolomics approaches have correlated SA and JA biosynthesis and signaling with FHB resistance (Li and Yen, 2008; Ding et al., 2011; Gottwald et al., 2012; Makandar et al., 2012; Sun et al., 2016). ET was also mentioned to have contrasted impacts, either neutral (Sun et al., 2016), preventing (Li and Yen, 2008) or promoting fungal infection (Chen et al., 2009). However, only few researches could fully demonstrate the involvement of phytohormones in cereals/*F. graminearum* interactions through reverse genetics approaches. Functional genetics studies performed using transgenic wheat lines or fungal strains expressing the *NahG* gene, have shown that the degradation of SA through the NahG salicylate hydroxylase activity increased disease severity (Makandar et al., 2012; Qi et al., 2019). Expression of the *A. thaliana NPR1* gene in wheat has further confirmed the involvement of SA signaling in FHB resistance (Makandar et al., 2006). No functional studies are available yet for JA biosynthesis or signaling. Chen et al. (2009) have shown that wheat *ein2*-silenced lines exhibited increased FHB resistance suggesting that *F. graminearum* may take advantage of the host ET signaling pathway for infection. In more recent studies, other phytohormones have been correlated either with FHB susceptibility (auxins, ABA) or with resistance (GAs) to FHB (Buhrow et al., 2016; Wang et al., 2018a). Such a picture exemplifies the complexity of hormonal crosstalk during the interaction, as already described in many pathosystems (Pieterse et al., 2009).

Two decades ago, SLs emerged as a new class of phytohormones (Al-Babili and Bouwmeester, 2015). Even more recently, the role of SLs in plant–pathogen interactions using biosynthesis or signaling mutants has been investigated. Mostly conducted in dicotyledonous plant species, these works have concluded on either no impact (Piisilä et al., 2015; Blake et al., 2016; Foo et al., 2016) or a role of SLs in resistance toward pathogenic microorganisms whatever their trophic habit (Torres-vera et al., 2014; Piisilä et al., 2015; Stes et al., 2015; Xu et al., 2019). To our knowledge, a single work on rice has described the impact of impaired SLs biosynthesis on the interaction with a plant pathogen (Lahari et al., 2019). In contrast with the results obtained on the tomato—*Meloidogyne incognita* interaction concluding on a role of SLs in resistance (Xu et al., 2019), Lahari et al. (2019) have shown that SLs increased rice susceptibility to the root-knot nematode *M. graminicola*, suggesting that differences may exist between dicots and monocots.

### *BdCYP711A29* Gene Is Induced by DON and Involved in Orobanchol Biosynthesis

In this work, we have functionally characterized a CYP-encoding gene from *B. distachyon*, *BdCYP711A29* (*Bradi1g75310*), previously identified as differentially induced in a transcriptomic study of the plant response to *F. graminearum*-produced DON during infection (Pasquet et al., 2014). Detailed analysis of the deduced amino-acid sequence (Supplementary Figure 1) revealed that the gene encodes the *B. distachyon* BdCYP711A29 protein of the CYP711A subfamily comprising four additional members in *B. distachyon* [BdCY711A5, A6, A30, and A31 (Figure 2)]. The BdCYP711A29 protein belongs to the same clade as the *A. thaliana* MAX1 protein involved in the conversion of CL into CLA (Abe et al., 2014) and as two *O. sativa* CYP711A proteins (OsCYP711A2 and A3) involved in SL biosynthesis downstream of CL (Cardoso et al., 2014; Zhang et al., 2014, Figure 2). We validated differential induction of *BdCYP711A29* expression following infection by a DON-producing *F. graminearum* strain compared to a mutant strain unable to produce the mycotoxin and also showed that expression was induced following direct application of the mycotoxin (Figure 1). We further showed that the expression of the four other *BdCYP711A* genes were either not or far less regulated in the same conditions (Supplementary Figure 4). The five members of the oligogenic family were differentially expressed in *B. distachyon* organs with *BdCYP711A5* being more expressed in leaves and spikes, *BdCYP711A6* and *A29* more expressed in roots and spikes whereas *BdCYP711A30* and *A31* are almost exclusively expressed in roots (Figure 3). These results suggested that, based on the expression of the five corresponding genes, the proteins may either play redundant roles (BdCYP711A6 and A29 or BdCYP711A30 and A31) or differential ones along the whole plant.

To investigate the role of BdCYP711A29 in SL biosynthesis, *B. distachyon* lines altered in *BdCYP711A29* gene expression or sequence were generated or selected. Two transgenic lines carrying a single T-DNA insertion and overexpressing the gene were generated as well as their corresponding null segregant (Figure 4 and Table 1). Using a TILLING mutant collection available in our laboratory (Dalmais et al., 2013), two mutant lines were selected both carrying mutations predicted to strongly impact the CYP functionality, together with their control lines carrying a WT *BdCYP711A29* allele (Table 1 and Supplementary Figure 2 and Table 4). Based on the role of SLs in plant development (Al-Babili and Bouwmeester, 2015), OE and mutant lines were characterized for shoot branching by measuring the tillers number and compared to the WT and their corresponding control lines. No statistically supported difference related to the alteration of the *BdCYP711A29* sequence or expression could be observed (Supplementary Figure 5) suggesting that BdCYP711A29 may not play *per se* a significant role in *B. distachyon* shoot development. Alternatively, functional redundancy with one of the four other CYP711A copies encoded by the *B. distachyon* genome could occur considering the expression patterns of the five genes (Figure 3).

Due to their very low amounts in plant tissues, SLs are difficult to quantify *in planta*. Nevertheless, when plants are grown on phosphate-deprived media, root exudation is stimulated allowing their detection and quantification (López-Ráez et al., 2008). To determine whether BdCYP711A29 is involved in SL biosynthesis, root exudates of the strongest overexpressing line OE-CYP12.20, the mutant line carrying a STOP codon M5374#135 and the WT line were extracted and relative quantification of SLs was performed. A 16-fold increase in orobanchol was observed in the OE line as compared to the WT line (Figure 5). Few studies have deeply investigated the role of MAX1-like proteins in SL biosynthesis and diversification. In rice, as in *B. distachyon*, five CYP711A proteins are found, all but one (Os1500) being functional (Zhang et al., 2014). Using *in vitro* co-expression studies, Zhang et al. (2014) have shown that among the four functional rice MAX1-like proteins, Os900 is a CL oxidase, catalyzing the conversion of CL into ent-22-epi-5-deoxystrigol whereas Os1400 catalyzes the conversion of the latter product into orobanchol. The BdCYP711A29 protein groups in the same clade as Os900 and Os1400, together with two other *B. distachyon* proteins, BdCYP711A30 and BdCYP711A31 (Figure 2). Altogether, our results strongly suggest that the BdCYP711A29 is involved in orobanchol biosynthesis. Additional experiments involving *in vitro* co-expression studies in yeast or in *Nicotiana benthamiana* leaves as described in Zhang et al. (2014) will determine the exact step catalyzed by the BdCYP711A29 protein.

### Overexpression of *BdCYP711A29* Increases Plant Susceptibility Toward FHB

Few CYPs have been shown to be involved in the interaction between cereals and *F. graminearum* and only two of them have been characterized as strongly associated with resistance to DON. Ito et al. (2013) identified a CYP from the Gram-negative bacterium *Sphingomonas* sp. able to convert DON into 16-hydroxy-deoxynivalenol. More recently, virus-induced gene silencing of wheat TaCYP72A copies demonstrated their involvement in resistance to DON and grain development (Gunupuru et al., 2018). Among the five *B. distachyon* CYP711A-encoding genes, *BdCYP711A29* is the only one strongly induced in spikes in a DON-dependent manner (Figure 1 and Supplementary Figure 4), suggesting a specific role in FHB. To assess the involvement of BdCYP711A29 in FHB, spikes of the different lines were spray-inoculated by a DON-producing *F. graminearum* strain and symptoms were scored 7 and 14 days after inoculation. Whereas no difference was observed between the WT ecotype Bd21-3, the null segregant NS-11-26 and the TILLING mutant lines or their corresponding control lines, the two OE lines, OE-CYP11.29 and OE-CYP12.20 were significantly more susceptible to FHB whatever the time-point (Figure 6). These results were confirmed by the quantification of fungal biomass by qPCR (Figure 7).

Strigolactones have been shown to be involved in complex cross-talks with other phytohormones (recently reviewed in Omoarelojie et al., 2019). In particular, tomato SL biosynthesis mutants were shown to have reduced JA content (Torres-vera et al., 2014). However, a more recent study in Arabidopsis has revealed no clear relationship between SLs and JA (Rozpadek et al., 2018). In contrast, in the same study, the authors have provided evidence of a complex crosstalk between SLs and SA: high doses of GR24 (1 μM) strongly induced SA biosynthesis in WT plantlets and *max1* mutants exhibited increased SA content (Rozpadek et al., 2018). Nevertheless, reports are not sufficient yet to fully decipher the relationships between SLs and other phytohormones in plant-biotic interactions. In our study, although no SA or JA quantification has been performed in the different lines, we could show that increased susceptibility is unlikely correlated to an alteration of plant defenses. Indeed, no major difference in the induction of defense marker genes frequently used in our team (Pasquet et al., 2014), could be observed (Figure 8). Even if we cannot rule out that some specific defense pathways may be altered in some lines, major changes in plant defense is therefore not the explanation for increased susceptibility of the OE lines.

### Orobanchol and Exudates of the *BdCYP711A29* Overexpressing Lines Stimulate *F. graminearum* Macroconidial Germination *in vitro*

Beyond their role *in planta*, SLs have been shown to have a direct impact on the development of fungal microorganisms (De Cuyper and Goormachtig, 2017; López-Ráez et al., 2017). *In vitro* studies using the synthetic analog GR24 provided contrasted results likely due to variation in experimental set ups rather than to species-specific differences. As an example, the impact of GR24 on the growth of the fungal ascomycete *B. cinerea* has been investigated in several studies but has led to contradictory results (Steinkellner et al., 2007; Dor et al., 2011; Torres-vera et al., 2014; Belmondo et al., 2017). Given the increased exudation of orobanchol by the OE line OE-CYP12.20, we have investigated the impact of a range of physiological concentrations of this SL on *F. graminearum*. We have shown that orobanchol concentrations from 10^–12^ to 10^–6^ M significantly stimulated germination of *F. graminearum* macroconidia as exemplified by an increased number of germ tubes (Figure 9A, Supplementary Figure 5). Using exudates recovered from phosphate-deprived hydroponic cultures of WT, *BdCYP711A29* overexpressing or mutant lines, we have further shown that *F. graminearum* macroconidial germination was significantly increased in root exudates of the two overexpressing lines (OE-CYP11.29 and OE-CYP12.20) whereas it was reduced in exudates of one of the mutant lines (M8687#12) compared to the WT line Bd21-3 (Figure 9B). Together with the increased orobanchol content found by LC-MS/MS in root exudates of the OE-CYP12.20 line, these results strongly support a role of this SL in the stimulation of germination of *F. graminearum* macroconidia. Such a role is highly reminiscent of the well-documented role of SLs in the stimulation of spore germination of AMF (García-Garrido et al., 2009). *F. graminearum* beyond its ability to infect cereal spikes, has been also shown to be a root pathogen (Wang et al., 2015). The impact of plant root exudates and more specifically of orobanchol on the stimulation of spore germination of this soil-borne fungus is therefore biologically relevant. In a recent study, Ding et al. (2020) have set up an experimental design to quantitatively assess *F. graminearum* root infection using *B. distachyon* as a model plant. We were however unable to reproduce this experimental set up and future experiments are needed to better correlate increased orobanchol in root exudates with *F. graminearum* aggressiveness on plant roots.

Considering spike infection, SL quantification in spikes of the different *B distachyon* lines has not been performed in this study due to the very low concentrations of SLs *in planta*. Nevertheless, the expression pattern of *BdCYP711A29* indicates both a higher basal expression in spikes as well as a strong induction of expression in the same organ at early steps of *F. graminearum* infection in a DON-dependent manner (Figures 1, 3). These results are therefore consistent with an involvement of this gene in the plant response during infection, likely through increasing orobanchol contents.

Altogether, the results obtained in this study strongly suggest that during the early steps *F. graminearum* infection the expression of the *B. distachyon BdCYP711A29* gene is induced which increases orobanchol production in its targeted organ. As a stimulant of spore germination, orobanchol may consequently promote infection and FHB development.

## Data Availability Statement

The original contributions presented in the study are included in the article/Supplementary Material, further inquiries can be directed to the corresponding author/s.

## Author Contributions

VC, SB-M, GM, and MDu conceived and designed the experiments. KM, MDa, and AB conducted the selection of TLLING mutants. VC, CM, SB-M, KM, GM, and MD conducted the experiments and analyses. VC and MD drafted the manuscript. All authors have read and approved the final version of the manuscript.

## Conflict of Interest

The authors declare that the research was conducted in the absence of any commercial or financial relationships that could be construed as a potential conflict of interest.

## Footnotes

1 http://tools.ips2.u-psud.fr/UTILLdb

2 http://drnelson.uthsc.edu/cytochromeP450.html

## Supplementary Material

Supplementary Figure 1 Nucleotide and deduced amino-acid sequence of the *BdCYP711A29* (*Bradi1g75310*) gene. The nucleotide sequence starts from the first line and the deduced protein sequence is indicated on the above line. Start and Stop codons as well as the initial methionine residue are indicated in bold. Introns are shaded in gray. The nucleotide sequences of primers used to amplify the region subjected to the TILLING screen are underlined. Amino-acid residues corresponding to the PERF signature and to the heme-binding domain are shown by blue and red letters, respectively.

Supplementary Figure 2 Schematic representation of the BdCYP711A29 protein (after Bak et al., 2011) and location of the mutations in TILLING families 8687 and 5374 (black arrows). The amplicon used for the selection of mutations in the *B. distachyon* TILLING mutant collection is indicated by a black bar.

Supplementary Figure 3 **(A)** Daughter profil of [M+H]+ 347 : orobanchol standard. **(B)** Chromatogram of MRM transitions in a typical sample and in the orobanchol standard.

Supplementary Figure 4 Expression pattern of the 5 *BdCYP711A* genes during FHB and following DON treatment. Relative quantification of *BdCYP711A5* (*Bradi3g08360*), *BdCYP711A6* (*Bradi1g37730*), *BdCYP711A30* (*Bradi4g08970*) and *BdCYP711A31* (*Bradi4g09040*) transcripts in the Bd21-3 (WT) ecotype of *B. distachyon* following DON treatment **(A)** or *F. graminearum* infection **(B)**. Timepoints are expressed in hours post-application (hpa, DON treatment) hours post-infection (hpi, *F*. *graminearum* infection). **(A)** Expression levels following DON treatment compared to mock treatment. **(B)** Expression levels following point infection with the *FgDON*^+^ strain of *F. graminearum* compared to mock treatment. The relative quantity of transcripts compared to mock condition was calculated using the comparative cycle threshold (Ct) method (2^–ΔΔ*Ct*^). The *B. distachyon UBC18* and *ACT7* genes (*Bradi4g00660* and *Bradi4g41850*) were used as endogenous controls to normalize the data for differences in input RNA between the different samples. Mean of three independent biological replicates ± standard deviation. Asterisks indicate significant differences with the “0” timepoint in each condition and for each gene (**p* value < 0.05, Student’s *t* test).

Supplementary Figure 5 Number of tillers developed by the different *B. distachyon* lines after 4 weeks growth under normal growth conditions (see section “Materials and Methods”). Mean numbers out of three biological replicates ± standard deviation. Different letters indicate significant differences between conditions (*p*-value < 0.01, Tukey’s test).

Supplementary Figure 6 Characteristic germination patterns of macroconidia of the *F. graminearum FgDON+* exposed at different orobanchol concentrations. **(A)** Control condition (0.01% DMSO), **(B)** 10^–6^ M orobanchol in 0.01% DMSO, **(C)** 10^–8^ M orobanchol in 0.01% DMSO, **(D)** 10^–10^ M orobanchol in 0.01% DMSO, **(E)** 10^–12^ M orobanchol in 0.01% DMSO. m: macroconidium, gt: germ tube. Bars: 100 μm.

Supplementary Table 1 List of *BdCYP711A29*-specific primers used for the construction of overexpressing lines and the selection of TILLING mutant lines.

Supplementary Table 2 List of primers used in qPCR experiments.

Supplementary Table 3 List of characteristic parent and product ions detected during multiple reaction monitoring (MRM) in *B. distachyon* (Bd21-3) exudates.

Supplementary Table 4 Pairwise distances matrix of of CYP711As from *B. distachyon, H. vulgare, O. sativa, A. thaliana and S. moellendorffii*. At: A. thaliana; Bd: B. distachyon; Hv: Hordeum vulgare; Os: O. sativa; Sm: S. moellendorffii. Protein sequences used in this analysis are available under the following accession numbers: AtCYP711A1, OAP07831.1; BdCYP711A5, XP_003571126.1; BdCYP711A6: XP_003560652.1; BdCYP711A29, XP_003562092.2; BdCYP711A30: XP_003575594.2; BdCYP711A31, XP_010237353.2; HvCYP711A5, BAJ97619.1; HvCYP711A6, KAE87888859.1; HVCYP711A29, BAJ98237.1; HvCYP711A30, KAE8810993.1; OsCYP711A2, XP_015633367.1; OsCYP711A3, XP_015644699.2; OsCYP711A4, XP_015642272.1; OsCYP711A5, XP_015626073.1; OsCYP711A6, XP_015644019.1; SmCYP711A1, XP_002972055.1. The protein mainly studied in this study is highlighted in orange, the protein sequence used as the outgroup uq highlighted in grey.

Supplementary Table 5 Characteristics of the mutant families identified in the *BdCYP711A29* gene following screening of the Bd21-3 TILLING collection (Dalmais et al., 2013).

Supplementary Table 6 Raw data and calculation of relative quantity of oranbanchol in exudates of WT (Bd21-3), overexpressing (OE-CYP12.20) and mutant (M5374#135) lines after 7 d phosphorus starvation. AUC: Area under the curve. MRM transitions 321 > 224 and 347 > 97 were used to quantify GR24 and orobanchol signals, respectively.
