# Supplementary material for: Overexpression of a Cytochrome P450 Monooxygenase Involved in Orobanchol Biosynthesis Increases Susceptibility to Fusarium Head Blight
Source: Front Plant Sci. 2021 Apr 1;12:662025. doi: 10.3389/fpls.2021.662025 (PMC8048717; doi:10.3389/fpls.2021.662025)
Supplement: Supplementary Figure 1 — Nucleotide and deduced amino-acid sequence of the BdCYP711A29 (Bradi1g75310) gene. The nucleotide sequence starts from the first line and the deduced protein sequence is indicated on the above line. Start and Stop codons as well as the initial methionine residue are indicated in bold. Introns are shaded in gray. The nucleotide sequences of primers used to amplify the region subjected to the TILLING screen are underlined. Amino-acid residues corresponding to the PERF signature and to the heme-binding domain are shown by blue and red letters, respectively. [file Data_Sheet_1.PDF]

```
CAAGACAGACGTACGATGGACGGAGCGGGAGTACTCTGGAGCCGTCAGATGGAGTCGCCATTGGCAGC
                                                M  E  S  P  L  A  A
GATTCTGTTCACCGTGGCGGCACTGGCGGCCGGCGCATTCGCCGTGTATTTCTACGCGCCGTCGTGGC
 I  L  F  T  V  A  A  L  A  A  G  A  F  A  V  Y  F  Y  A  P  S  W  R
GCCTGCGCAGGGTGCCCGGGCCTCTCGCCTACGGGCTCATCGGCCACCTGCCGCTCTTCACCAAGCAT
  L  R  R  V  P  G  P  L  A  Y  G  L  I  G  H  L  P  L  F  T  K  H
GGCCCCGAGGTCTTTGGCGTCCTCGCAAGGAGATACGGGCCCATCTACAGGTCAGTTGTTAATTAGTA
G  P  E  V  F  G  V  L  A  R  R  Y  G  P  I  Y  R
ACTCCACACACCGGCCGATCAATTTCAATTGGGTGGCTATATCCTACGTATATACTTGATTTTTCATC
TCACAGATCAACTTGTCCTAACTAAAAATTAAACGACTTAGATATATCAAACCTGTATCAGTTGTAGT
TTAAGGCTTTCGATCTGCACTTAATACGCTGGCGGTCGGTGTGTTCTGTCCAGGTTTTACCTGGGTCG
                                                      F  Y  L  G  R
ACAGCCGGTGGTGGTGATCGCGGACGCAGAGCTGTGCAGGGAGGCCGGCATCAAGAAGTTCAAGAGCG
 Q  P  V  V  V  I  A  D  A  E  L  C  R  E  A  G  I  K  K  F  K  S  V
TCGTCGACAGGAGCGTGCCCAGCACCATCCGTAGCTCGCCCATCCACTTCAAAAGTCTCCTCTTCACC
  V  D  R  S  V  P  S  T  I  R  S  S  P  I  H  F  K  S  L  L  F  T
AAGTACGTCCAACTTACAATGGAGATCGCGTCTTGGACATAATTAAGCTGCTAGAGCTAGCTAGAGCT
K
AGTTTTAAACGCATGCAAACCAAATTAATTGCAGGGGCTCGAGGTGGCAGTCGATGCGGAACGTGATC
                                   G  S  R  W  Q  S  M  R  N  V  I
ATCGCCATCTACCAGCCATCTCACCTTGCAAGCCTCATCCCGGCCGTCCATCCCTACATCCGCCGCGC
I  A  I  Y  Q  P  S  H  L  A  S  L  I  P  A  V  H  P  Y  I  R  R  A
CGCCCGTCTCCTCCACCCCGGCCAAGAGGTCGCCTTCTCCGACCTCGCCGTCAAGCTCTTCTCCGACA
 A  R  L  L  H  P  G  Q  E  V  A  F  S  D  L  A  V  K  L  F  S  D  T
CCATCGGGCAAGCCGCGTTCGGCGTCGACTTCGGGCTCACCAAACCCGACGACGCCAACAATGTCGAC
  I  G  Q  A  A  F  G  V  D  F  G  L  T  K  P  D  D  A  N  N  V  D
AGCACCATCAACAACGAAAAAACAGCCACGGACGACTTCATCGAGAAGCACCTGTACGCGCTGACGTC
S  T  I  N  N  E  K  T  A  T  D  D  F  I  E  K  H  L  Y  A  L  T  S
GCTCAAGGCGGACCTCAATGGGTCGCTCTCCATGGTGCTGGGAACCGTGGCGCCGCTGCTGCAGGAGC
 L  K  A  D  L  N  G  S  L  S  M  V  L  G  T  V  A  P  L  L  Q  E  P
CGGCCCGGCAGCTGCTGCTGCGGGTGCCGGGCTCGGCGGACCGCCTGATGGACGAGACCAACCGGGCC
  A  R  Q  L  L  L  R  V  P  G  S  A  D  R  L  M  D  E  T  N  R  A
CTTAGCGGGCTCGTGGACGCCATCGTGGCGGAGCGGGCCGCCATGGAGGCCCAGAGCGAGGGGGAGAA
L  S  G  L  V  D  A  I  V  A  E  R  A  A  M  E  A  Q  S  E  G  E  K
GAAGAACTTCCTGTCCGTGCTGCTCAAGGCCCGGGAGAGCAGCCACGCCATGAGGGAGCTCTTCACGG
 K  N  F  L  S  V  L  L  K  A  R  E  S  S  H  A  M  R  E  L  F  T  A
CCGACTACGTCTCCGCGCTCACCTACGAGCACTTGCTCGCCGGGTCCGGGTCCATGTCCTTCACGCTC
  D  Y  V  S  A  L  T  Y  E  H  L  L  A  G  S  G  S  M  S  F  T  L
TCGGGACTCGCGTACCGGGTCGCCATGCACCCGGAGGTGGAGGAGAAGATGCTAAGTGAGATCGACGC
S  G  L  A  Y  R  V  A  M  H  P  E  V  E  E  K  M  L  S  E  I  D  A
GTTTGGGCCCAAGGACCTCGTGCCTGACGCCGAGGAACTCAACACCAAGTTCACCTACTTGGAGCAGG
 F  G  P  K  D  L  V  P  D  A  E  E  L  N  T  K  F  T  Y  L  E  Q
TTCGTCTCGGTCGTTTGCACGATCTTCAGATTAAACGTATCGATTCCCTAAAAAAAAAGATTAAACGT
ATCGATCATAACCTGTGTTAACATCCATGTACAACTTTTGCCCCAATAGGTTTTGAAGGAGACGATGA
                                                 V  L  K  E  T  M  R
GGTTCTATTCTTCGTCCCCGCTGGTTTCGAGGGAAACGACCGAGGACGTCGAGATCGGAGGCTATCTC
  F  Y  S  S  S  P  L  V  S  R  E  T  T  E  D  V  E  I  G  G  Y  L
CTGCCAAAGGTTCGTGCCAATCTGCTGCCTTATGTGGTCAAGGCTAAAATGGAATATGTACGGCCACA      CYP711A29-F1
L  P  K
TCAATTTTCATTTTTATGAGTTTACCGAGTCAAAAATGGTGCCTGCACGCACGCAGGGAACGTGGGTG
                                                        G  T  W  V
TGGCTAGCGACGGGGCAGCTATCGAAAGACCCGAAGCATTTCCCCGACCCGTACACGTTCCGTCCCGA
W  L  A  T  G  Q  L  S  K  D  P  K  H  F  P  D  P  Y  T  F  R  P  E
GCGGTTCGACCCGGAGGACGAGGAATGCAAGCGAAGGCACCCCTACGCGTTCCTCCCCTTCGGCATCG
 R  F  D  P  E  D  E  E  C  K  R  R  H  P  Y  A  F  L  P  F  G  I  G
GCCCTCGGGGTTGCCCCGGGCAGAAGTTCGCCATGCAGCAGCTCAAGCTCGTGGTTATCCACCTGTAC
  P  R  G  C  P  G  Q  K  F  A  M  Q  Q  L  K  L  V  V  I  H  L  Y
CGCCGCTACGTCTTCAGGCACTCCCCCGGCATGGAGTTTCCCCTGCAGCTCGAGTTCTCCATCGTGAA
R  R  Y  V  F  R  H  S  P  G  M  E  F  P  L  Q  L  E  F  S  I  V  N
CAACTTCAAGCATGGCGTCAAGCTCCAAGTCATCGATCGAGAAGAGCACTAACTGATGTACTCCGTGT      CYP711A29-R
 N  F  K  H  G  V  K  L  Q  V  I  D  R  E  E  H  *
GA
```

Supplementary Figure 1: Nucleotide and deduced amino-acid sequence of the *BdCYP711A29* (*Bradi1g75310*) gene. The nucleotide sequence starts from the first line and the deduced protein sequence is indicated on the above line. Start and Stop codons as well as the initial methionine residue are indicated in bold. Introns are shaded in gray. The nucleotide sequences of primers used to amplify the region subjected to the TILLING screen are underlined. Amino-acid residues corresponding to the PERF signature and to the heme-binding domain are shown by blue and red letters, respectively.

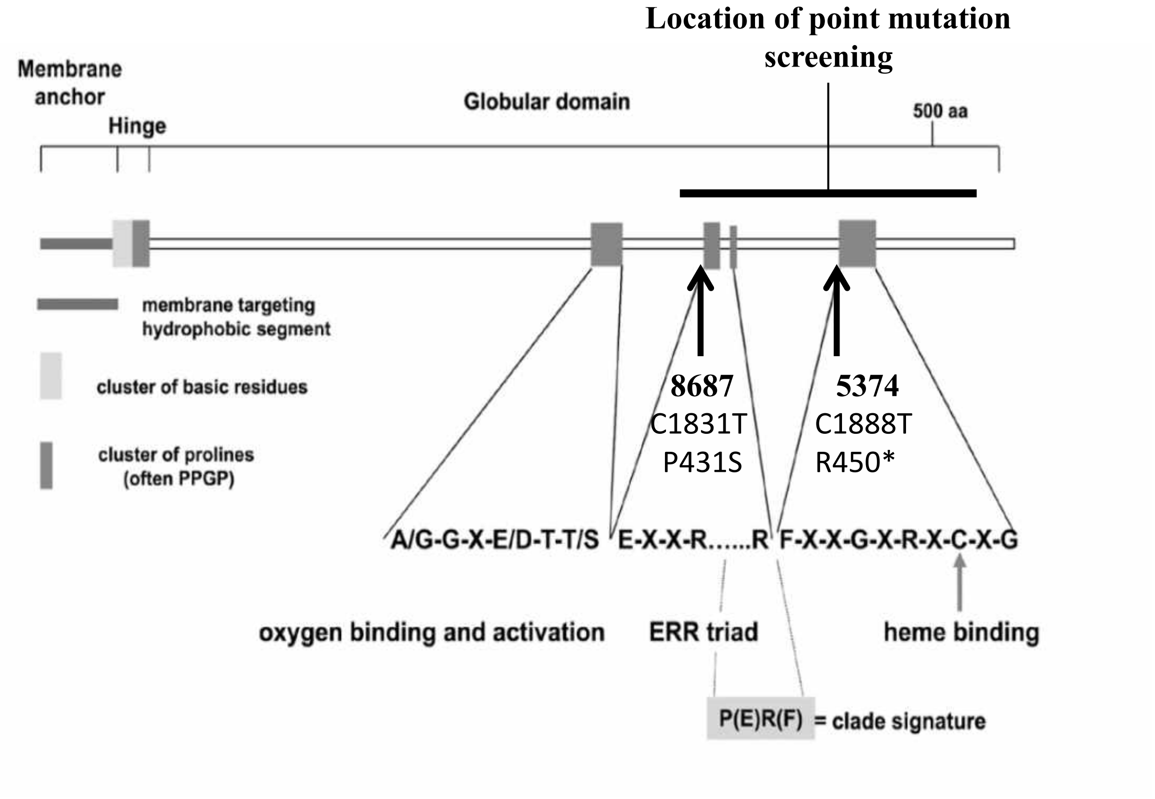

Supplementary Figure 2: Schematic representation of the BdCYP711A29 protein (after Bak et al., 2011) **and location of the mutations in TILLING families 8687 and 5374** (black arrows). The amplicon used for the selection of mutations in the *B. distachyon* TILLING mutant collection is indicated by a black bar. For each mutant family, the nucleotide transition and amino-acid substitution are indicated below the family number. The asterisk stands for a STOP codon.

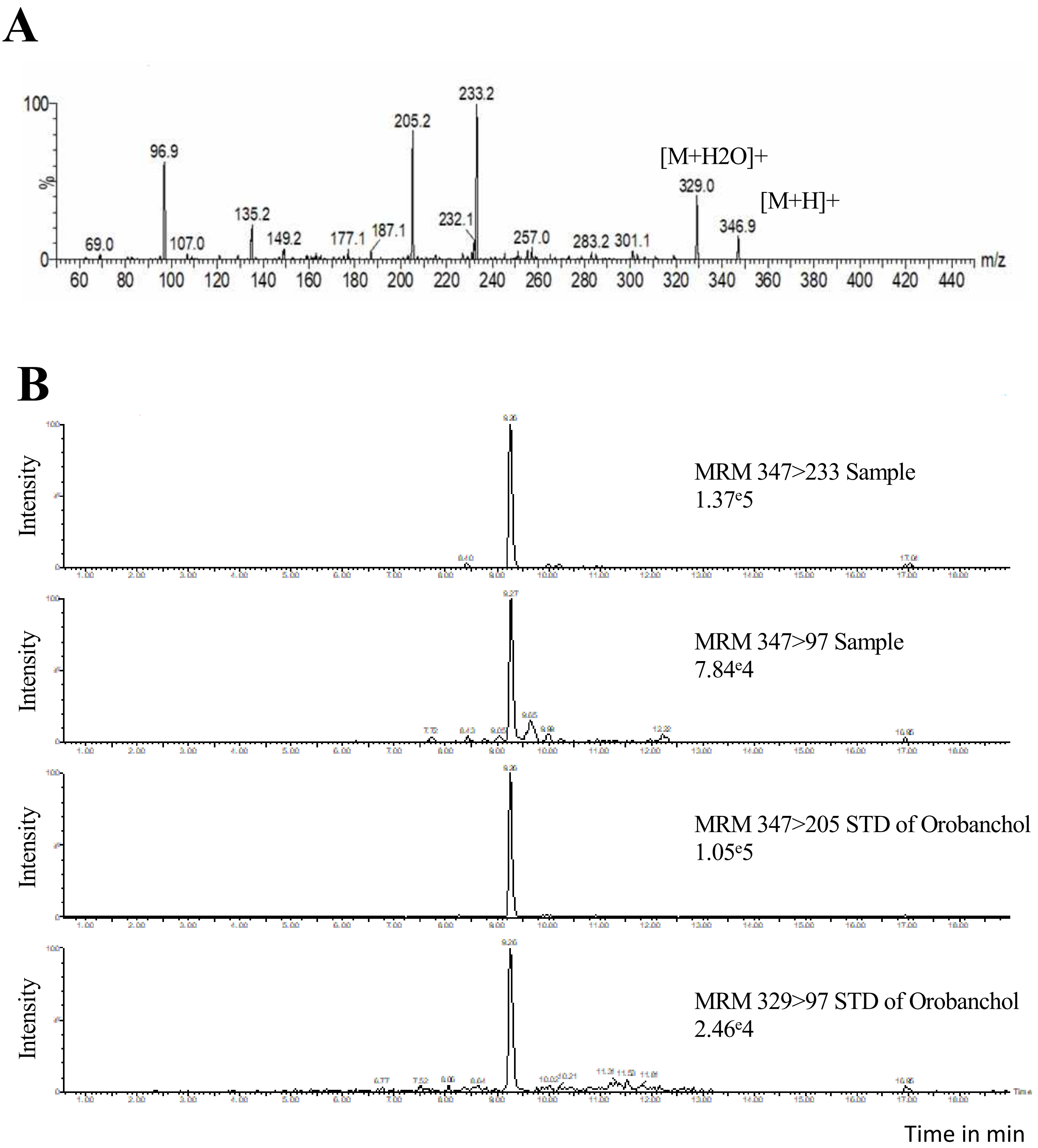

Supplementary Figure 3: A. Daughter profil of [M+H]+ 347 : orobanchol standard. **B.** Chromatogram of MRM transitions in a typical sample and in the orobanchol standard.

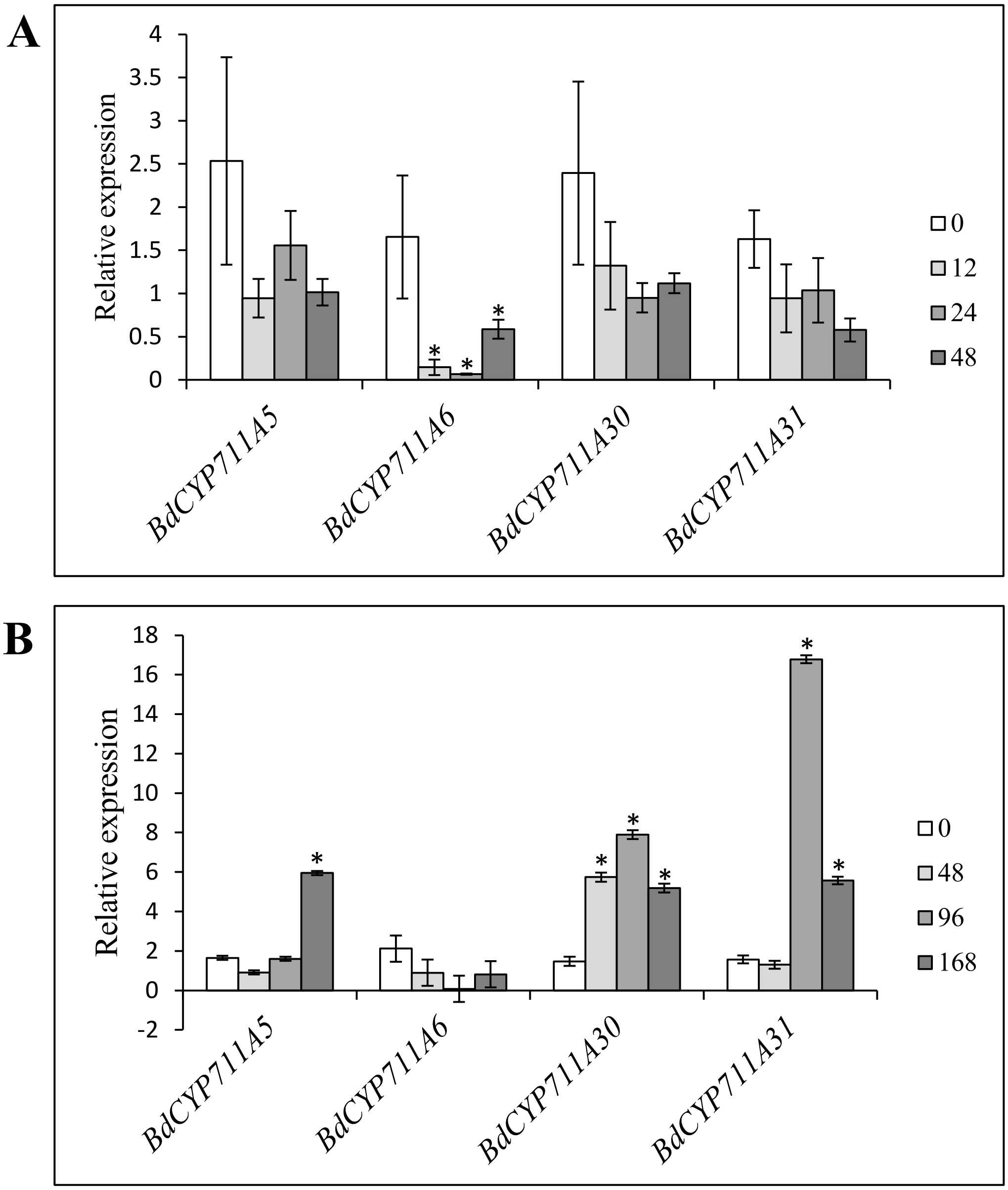

Supplementary Figure 4: Expression pattern of the four other *BdCYP711A* genes following DON treatment and during FHB. Relative quantification of *BdCYP711A5* (*Bradi3g08360*), *BdCYP711A6* (*Bradi1g37730*), *BdCYP711A30* (*Bradi4g08970*) and *BdCYP711A31* (*Bradi4g09040*) transcripts in the Bd21-3 (WT) ecotype of *B. distachyon* following DON treatment (**A**) or *F. graminearum* infection (**B**). Timepoints are expressed in hours post-application (hpa, DON treatment) hours post-infection (hpi, *F. graminearum* infection). **A**. Expression levels following DON treatment compared to mock treatment. **B**. Expression levels following point infection with the *FgDON*$^{+}$ strain of *F. graminearum* compared to mock treatment. The relative quantity of transcripts compared to mock condition was calculated using the comparative cycle threshold (Ct) method ($2^{-\Delta\Delta Ct}$). The *B. distachyon UBC18* and *ACT7* genes (*Bradi4g00660* and *Bradi4g41850*) were used as endogenous controls to normalize the data for differences in input RNA between the different samples. Mean of three independent biological replicates ± standard deviation. Asterisks indicate significant differences with the "0" timepoint in each condition and for each gene (*p value < 0.05, Student's t test).

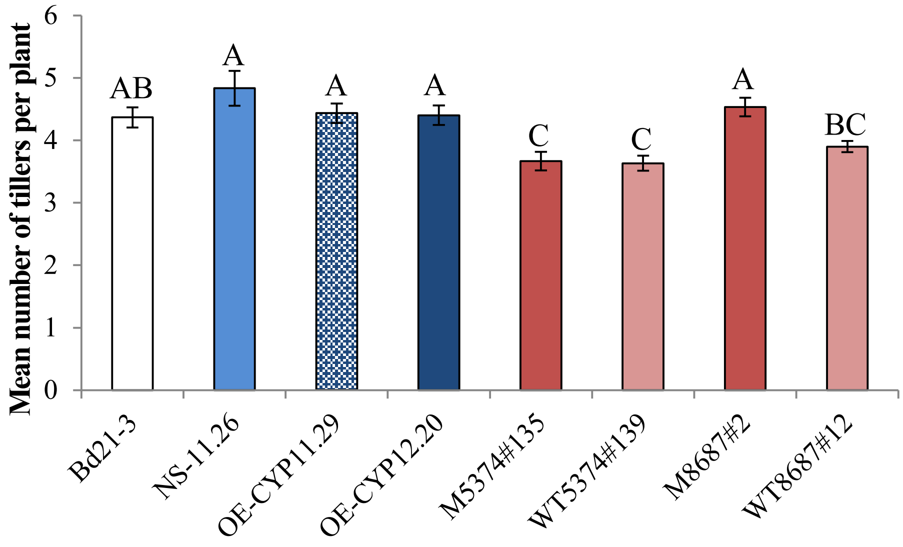

Supplementary Figure 5: Number of tillers developed by the different *B. distachyon* lines after 4 weeks growth under normal growth conditions (see Material and Methods). Mean numbers out of three biological replicates ± standard deviation. Different letters indicate significant differences between conditions (p-value < 0.01, Tukey test).

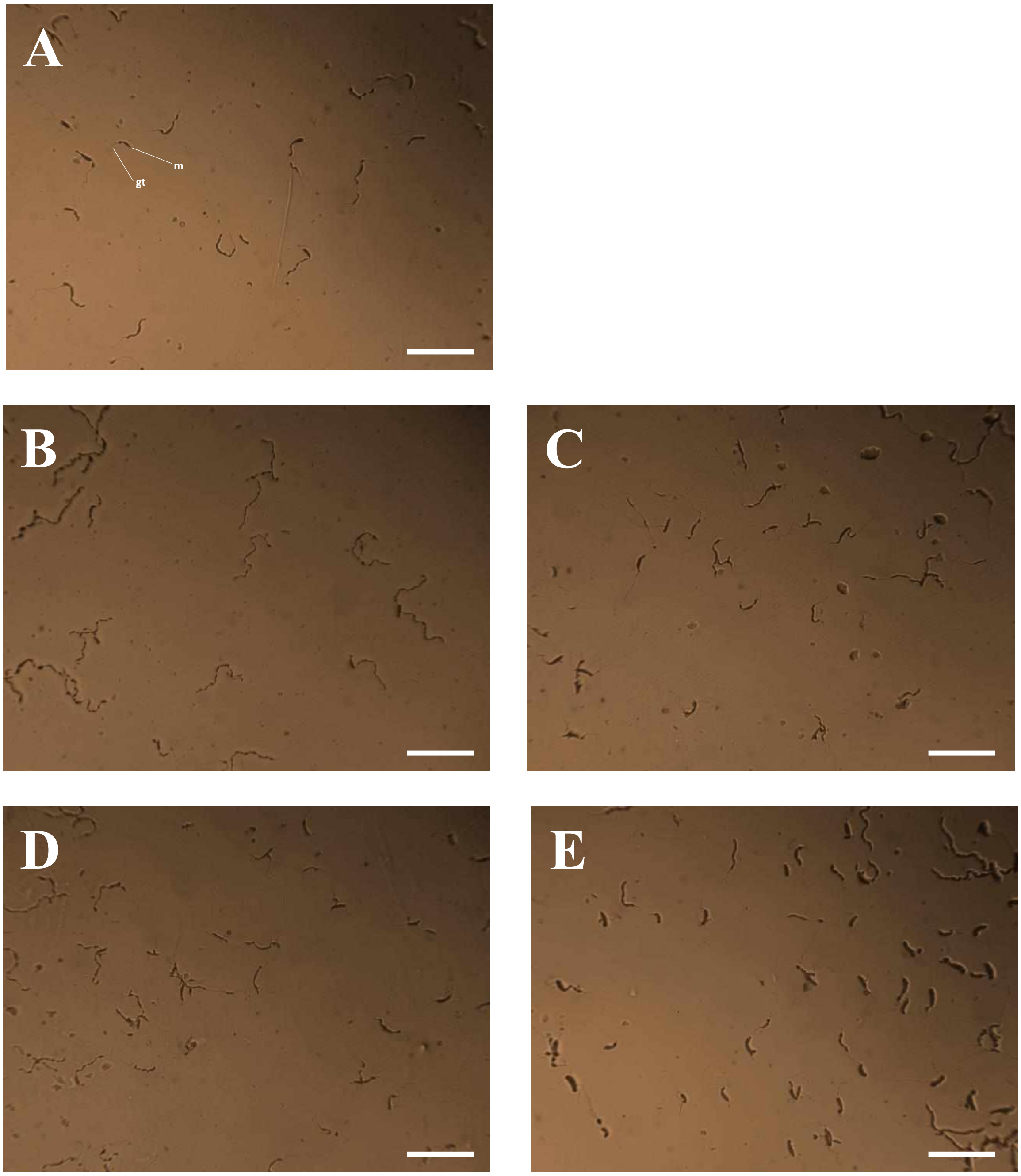

Supplementary Figure 6: Characteristic germination patterns of macroconidia of the *F. graminearum FgDON+* exposed at different orobanchol concentrations. (A) Control condition (0.01% DMSO), (B) 10^{-6} M orobanchol in 0.01% DMSO, (C) 10^{-8} M orobanchol in 0.01% DMSO, (D) 10^{-10} M orobanchol in 0.01% DMSO, (E) 10^{-12} M orobanchol in 0.01% DMSO. m: macroconidium, gt: germ tube. Bars: 100 µm.
