# Supplementary material for: Overexpression of a Cytochrome P450 Monooxygenase Involved in Orobanchol Biosynthesis Increases Susceptibility to Fusarium Head Blight
Source: Front Plant Sci. 2021 Apr 1;12:662025. doi: 10.3389/fpls.2021.662025 (PMC8048717; doi:10.3389/fpls.2021.662025)
Supplement: Supplementary Table 1 — List of BdCYP711A29-specific primers used for the construction of overexpressing lines and the selection of TILLING mutant lines. [file Data_Sheet_2.PDF]

**Supplementary Table 1.** List of *BdCYP711A29*-specific primers used for the construction of overexpressing lines and the selection of TILLING mutant lines**.**

| Primer name | Use | Polarity | Nucleotide sequence | Amplicon size (bp) | Reference |
|---|---|---|---|---|---|
| Bd1g75310_BI5'ATG[†] | Cloning | Forward | 5'-GAGGATCCATGGAGTCGCCATTGG-3' | 2347 (gDNA) / 1621 (cDNA) | This study |
| Bd1g75310_EV3'TAA[†] | | Reverse | 5'-CCGATATCTTAGTGCTCTTCTCGATCG-3' | | |
| CYP711A29-F1[‡] | TILLING | Forward | 5'-ttccctacacgacgctcttccgatctcGGCCACATCAATTTTCATT-3' | 452 (gDNA) | This study |
| CYP711A29-R[‡] | | Reverse | 5'-agttcagcgtgtgtcttccgatctTTAGTGCTCTTCTCGATCGA-3' | | |

[†], underlined letters correspond to the added restriction sites (*Bam*HI for Bd1g75310_BI5'ATG and *Eco*RV for Bd1g75310_EV3'TAA, respectively).

[‡], small-case letters correspond to Illumina adapters sequences.

**Supplementary Table 2.** List of primers used in qPCR experiments**.**

| Gene | Nucleotide sequence | Polarity | Putative function | Reference |
|---|---|---|---|---|
| *Bradi4g00660* | 5'-ACCCTCTACGCTGGTGAGAC-3'<br>5'-TTGCTGTAAATGTGCGGATG-3' | Forward<br>Reverse | UBC18 (plant reference gene) | Pasquet et al. (2014) |
| *Bradi4g41850* | 5'-CCTGAAGTCCTTTTCCAGCC-3'<br>5'-AGGGCAGTGATCTCCTTGCT-3' | Forward<br>Reverse | ACT3-like (plant reference gene) | Pasquet et al. (2014) |
| *BdCYP711A5* | 5'- TTCGGCCCTCAATCTCAATCC-C-3'<br>5'-TGGTCCAGGTACGGGAATTTCG-3' | Forward<br>Reverse | Cytochrome P450 monoxygenase | This study |
| *BdCYP711A6* | 5'-TGCACATGTACCGGAGATTCGTG-3'<br>5'-ATTGCGCGTCAGCCTCTTGATG-3' | Forward<br>Reverse | Cytochrome P450 monoxygenase | This study |
| *BdCYP711A29* (*Bradi1g75310*) | 5'- GCTACGTCTTCAGGCACTCC-3'<br>5'- CGATCGATGACTTGGAGCTT-3' | Forward<br>Reverse | Cytochrome P450 monoxygenase | This study |
| *BdCYP711A30* | 5'-TACATCTTCCGCCACTCTCC-3'<br>5'-TTGAGGGGATCGATGTCTGG-3' | Forward<br>Reverse | Cytochrome P450 monoxygenase | This study |
| *BdCYP711A31* | 5'-CAACTTCAAGCACGGTGTCA-3'<br>5'-TATTGTACGCCACTTGTCGC-3' | Forward<br>Reverse | Cytochrome P450 monoxygenase | This study |
| *BdMAX3* | 5'- ATGTCGCCAATGGGTCAACG-3'<br>5'- TTTGTCTTCGGATACCGCATACTC-3' | Forward<br>Reverse | CCD7-like | This study |
| *BdMAX4* | 5'-TCGTCTCTGCTTCTTGCATACTGG-3'<br>5'-GCTGCACAAACATCCACGTACAG-3' | Forward<br>Reverse | CCD8-like | This study |
| *Bradi1g57590* | 5'-CAGGTGGTGTGGAGGAAGTC -3'<br>5'-CTCTCCCCGTTGAAGTTCCC -3' | Forward<br>Reverse | Pathogenesis-related protein 1-5 | Kouzai et al. (2016) |
| *Bradi1g39190* | 5'-TCCGACCAGGCTCTCTAC-3'<br>5'-GGTATGTTCCCCATCTTGAC-3' | Forward<br>Reverse | Pathogenesis-related protein PR9 | Pasquet et al. (2014) |
| *Bradi3g47110* | 5'-CCAAACAATTAAGGAGATCAATTAGAA-3'<br>5'- CCCGAATACTGGAAAGTAAGATACA-3' | Forward<br>Reverse | Phenylalanine ammonia-lyase | Pasquet et al. (2014) |
| 18S *F. graminearum* | 5'-GTCCGGCCGGGCCTTTCC-3'<br>5'-AAGTCCTGTTTCCCCGCCACGC-3' | Forward<br>Reverse | Ribosomal region 18S (fungal reference gene) | Mudge et al. (2006) |

**Supplementary Table 3:** List of characteristic parent and product ions detected during multiple reaction monitoring (MRM) in *B. distachyon* (Bd21-3) exudates.

| Compounds (parent ion form) detected in MRM | *m/z* Parent ion | *m/z* Daughter ion | RT (min) |
|---|---|---|---|
| 4-deoxyorobanchol [M + Na] [a] | 353 | 241 | 4,24 |
| 5-deoxystrigol [M + H] [b, c, d, *]/ Unknown 5 from Maize [M - OH] [b, *] | 331 | 97 | 8,56 |
| 5-deoxystrigol [M + H] [b, c, d, *] / Unknown 5 from Maize [M - OH] [b, *] | 331 | 97 | 9,52 |
| Carlactonoic acid [M + H] [b, *] | 333 | 97 | 8,91 |
| DidehydroOrobanchol [M + Na] [*] / DidehydroStrigol [M + H] [*] | 345 | 97 | 7,36 |
| DidehydroOrobanchol [M + Na] [*] / DidehydroStrigol [M + H] [*] | 345 | 248 | 9,64 |
| DidehydroOrobanchol [M + Na] [e, *] / DidehydroStrigol [M + Na] [e, *] | 367 | 270 | 6,03 |
| Hydroxyorobanchol [M + Na] [f, *] | 385 | 288 | 9,2 |
| Methoxy-5-Deoxystrigol [M + H] [g, *] / Heliolactone [M + H] [b, d, *] / Unknown 6 from Maize [M + H] [b, *] | 361 | 97 | 10,67 |
| Orobanchol [M - $H_2O$] [*] | 329 | 97 | 9,2 |
| Orobanchol [M - $H_2O$] [*] / Strigol [M - $H_2O$] [c, d, *] / Sorgomol [M - $H_2O$] [*] | 329 | 97 | 7,41 |
| Orobanchol [M + H] [c, d, h, i, m] | 347 | 205 | 9,19 |
| Orobanchol [M + H] [c, d, h, m, *] | 347 | 97 | 9,2 |
| Orobanchol [M + H] [d, h, i, j] | 347 | 233 | 9,2 |
| Orobanchyl acetate (Alectrol) [M + H] [h, m] | 389 | 347 | 8,05 |
| Orobanchyl acetate (Alectrol) [M + H] [c, d, h, k, m] | 389 | 233 | 8,9 |
| Solanacol [M + H] [*] | 343 | 246 | 9,64 |
| Solanacol [M + Na] [e, n, *] | 365 | 268 | 7,02 |
| Solanacyl acetate [M + H] [c, *] | 325 | 97 | 4,15 |
| Solanacyl acetate [M + H] [c] | 325 | 279 | 11,51 |
| Sorgomol [M + H] [c] / Strigol [M + H] [c] | 347 | 215 | 7,49 |
| Sorgomol [M + Na] [e, l, *] / Strigol [M + Na] [d, *] | 369 | 272 | 5,45 |
| Strigol [M - $H_2O$] [c] | 329 | 215 | 5,89 |
| Unknown 4 from Maize [M - OH] [b] | 375 | 343 | 10,94 |
| Zealactones [M + H] [b] / Unknown 2 from Maize [M + H] [b] | 377 | 345 | 9,14 |

Each transition has been detected at least in 1 out of 3 independent experiments. The three orobanchol transitions in grey have been validated using a standard in a preliminary experiment.* SL specific transition: detection or release of a *m/z* 97 fragment. [a] Boari et al. (2016), [b] Charnikhova et al. (2017), [c] Xie et al. (2015), [d] Iseki et al. (2018), [e] Yoneyama et al. (2008), [f] Khetkam et al. (2014), [g] Cardoso et al. (2014), [h] Kohlen et al. (2011), [i] Boutet-Mercey et al. (2018), [j] Foo & Davies (2011), [k] Kohlen et al., (2013), [l] Yoneyama et al. (2010), [m] Pavan et al. (2016), [n] Xie et al. (2013).

References


Boari, A., Ciasca, B., Pineda-Martos, R., Lattanzio, V. M., Yoneyama, K., & Vurro, M. (2016). Parasitic weed management by using strigolactones-degrading fungi. *Pest Management Science*, (November 2015), n/a-n/a. https://doi.org/10.1002/ps.4226

Boutet-Mercey, S., Perreau, F., Roux, A., Clavé, G., Pillot, J. P., Schmitz-Afonso, I., … Boyer, F. D. (2018). Validated Method for Strigolactone Quantification by Ultra High-Performance Liquid Chromatography – Electrospray Ionisation Tandem Mass Spectrometry Using Novel Deuterium Labelled Standards. *Phytochemical Analysis*, *29*(1), 59–68. https://doi.org/10.1002/pca.2714

Cardoso, C., Zhang, Y., Jamil, M., Hepworth, J., Charnikhova, T., Dimkpa, S. O. N., … Ruyter-Spira, C. (2014). Natural variation of rice strigolactone biosynthesis is associated with the deletion of two *MAX1* orthologs. *Proceedings of the National Academy of Sciences*, *111*(6), 2379–2384. https://doi.org/10.1073/pnas.1317360111

Charnikhova, T. V, Gaus, K., Lumbroso, A., Sanders, M., Vincken, J.-P., De Mesmaeker, A., … Bouwmeester, H. J. (2017). Zealactones. Novel natural strigolactones from maize. *Phytochemistry*, *137*, 123–131. https://doi.org/10.1016/j.phytochem.2017.02.010

Dalmais, M., Antelme, S., Ho-Yue-Kuang, S., Wang, Y., Darracq, O., d'Yvoire, M. B., … Sibout, R. (2013). A TILLING Platform for Functional Genomics in Brachypodium distachyon. *PLoS ONE*, *8*(6). https://doi.org/10.1371/journal.pone.0065503

Foo, E., & Davies, N. W. (2011). Strigolactones promote nodulation in pea. *Planta*, *243*, 1073–1081. https://doi.org/10.1007/s00425-011-1516-7

Iseki, M., Shida, K., Kuwabara, K., Wakabayashi, T., Mizutani, M., Takikawa, H., & Sugimoto, Y. (2018). Evidence for species-dependent biosynthetic pathways for converting carlactone to strigolactones in plants. *Journal of Experimental Botany*, *69*(9), 2305–2318. https://doi.org/10.1093/jxb/erx428

Khetkam, P., Xie, X., Kisugi, T., Kim, H. Il, Yoneyama, K., Uchida, K., … Yoneyama, K. (2014). 7α- and 7β-Hydroxyorobanchyl acetate as germination stimulants for root parasitic weeds produced by cucumber. *Journal of Pesticide Science*, *39*(3), 121–126. https://doi.org/10.1584/jpestics.D14-038

Kohlen, W., Charnikhova, T., Bours, R., Lopez-Raez, J. A., & Bouwmeester, H. (2013). Tomato strigolactones: a more detailed look. *Plant Signaling & Behavior*, *8*(1), e22785. https://doi.org/10.4161/psb.22785

Kohlen, W., Charnikhova, T., Liu, Q., Bours, R., Domagalska, M. A., Beguerie, S., … Ruyter-Spira, C. (2011). Strigolactones are transported through the xylem and play a key role in shoot architectural response to phosphate deficiency in nonarbuscular mycorrhizal host Arabidopsis. *Plant Physiology*, *155*(2), 974–987. https://doi.org/10.1104/pp.110.164640

Kouzai, Y., Kimura, M., Yamanaka, Y., Watanabe, M., Matsui, H., Yamamoto, M., … Noutoshi, Y. (2016). Expression profiling of marker genes responsive to the defence-associated phytohormones salicylic acid, jasmonic acid and ethylene in Brachypodium distachyon. *BMC Plant Biology*, *16*(1), 1–11. https://doi.org/10.1186/s12870-016-0749-9

Mudge, A. M., Dill-Macky, R., Dong, Y., Gardiner, D. M., White, R. G., & Manners, J. M. (2006). A role for the mycotoxin deoxynivalenol in stem colonisation during crown rot disease of wheat caused by Fusarium graminearum and Fusarium pseudograminearum. *Physiological and Molecular Plant Pathology*, *69*(1–3), 73–85. https://doi.org/10.1016/j.pmpp.2007.01.003

Pasquet, J. C., Chaouch, S., Macadré, C., Balzergue, S., Huguet, S., Martin-Magniette, M. L., … Dufresne, M. (2014). Differential gene expression and metabolomic analyses of Brachypodium distachyon infected by deoxynivalenol producing and non-producing strains of Fusarium graminearum. *BMC Genomics*, *15*(1), 1–17. https://doi.org/10.1186/1471-2164-15-629

Pavan, S., Schiavulli, A., Marcotrigiano, A. R., Bardaro, N., Bracuto, V., Ricciardi, F., … Ricciardi, L. (2016). Characterization of Low-Strigolactone Germplasm in Pea (Pisum sativum L.) Resistant to Crenate Broomrape (Orobanche crenata Forsk.). *Molecular Plant-Microbe Interactions : MPMI*, *29*(10), 743–749. https://doi.org/10.1094/MPMI-07-16-0134-R

Xie, X., Yoneyama, K., Kisugi, T., Nomura, T., Akiyama, K., Asami, T., & Yoneyama, K. (2015). Strigolactones are transported from roots to shoots, although not through the xylem. *Journal of*

*Pesticide Science*, *40*(4), 214–216. https://doi.org/10.1584/jpestics.D15-045

Xie, X., Yoneyama, K., Kisugi, T., Uchida, K., Ito, S., Akiyama, K., … Yoneyama, K. (2013). Confirming stereochemical structures of strigolactones produced by rice and tobacco. *Molecular Plant*, *6*(1), 153–163. https://doi.org/10.1093/mp/sss139

Yoneyama, K., Awad, A. A., Xie, X., Yoneyama, K., & Takeuchi, Y. (2010). Strigolactones as Germination Stimulants for Root Parasitic Plants. *Plant and Cell Physiology*, *51*(7), 1095–1103. https://doi.org/10.1093/pcp/pcq055

Yoneyama, K., Xie, X., Sekimoto, H., Takeuchi, Y., Ogasawara, S., Akiyama, K., … Yoneyama, K. (2008). Strigolactones, host recognition signals for root parasitic plants and arbuscular mycorrhizal fungi, from Fabaceae plants. *The New Phytologist*, *179*(2), 484–494. https://doi.org/10.1111/j.1469-8137.2008.02462.x

**Supplementary Table 4. Protein identity matrix based on ClustalW alignment of CYP711A protein sequences from *B. distachyon, H. vulgare, O. sativa, A. thaliana and S. moellendorffii* .** At: *A. thaliana* ; Bd: *B. distachyon* ; Hv: *Hordeum vulgare* ; Os: *O. sativa* ; *Sm: S. moellendorffii.* Protein sequences used in this analysis are available under the following accession numbers: AtCYP711A1, OAP07831.1; BdCYP711A5, XP_003571126.1; BdCYP711A6: XP_003560652.1; BdCYP711A29, XP_003562092.2; BdCYP711A30: XP_003575594.2; BdCYP711A31, XP_010237353.2; HvCYP711A5, BAJ97619.1; HvCYP711A6, KAE87888859.1; HVCYP711A29, BAJ98237.1; HvCYP711A30, KAE8810993.1; OsCYP711A2, XP_015633367.1; OsCYP711A3, XP_015644699.2; OsCYP711A4, XP_015642272.1; OsCYP711A5, XP_015626073.1; OsCYP711A6, XP_015644019.1; SmCYP711A1, XP_002972055.1. Values are indivated in percentages.

| | HvCYP711A30 | HvCYP711A6 | HvCYP711A29_ HvMAX1 | HvCYP711A5 | AtCYP711A1_ AtMAX1 | OsCYP711A2 | OsCYP711A3 | OsCYP711A4 | OsCYP711A5 | OsCYP711A6 | BdCYP711A5 | BdCYP711A6 | BdCYP711A29 | BdCYP711A30 | BdCYP711A31 | SmCYP711A1 |
|---|---|---|---|---|---|---|---|---|---|---|---|---|---|---|---|---|
| **HvCYP711A30** | 100.00 | | | | | | | | | | | | | | | |
| **HvCYP711A6** | 58.00 | 100.00 | | | | | | | | | | | | | | |
| **HvCYP711A29_HvMAX1** | 58.93 | 56.55 | 100.00 | | | | | | | | | | | | | |
| **HvCYP711A5** | 51.34 | 56.86 | 48.42 | 100.00 | | | | | | | | | | | | |
| **AtCYP711A1_AtMAX1** | 61.02 | 58.00 | 56.71 | 53.18 | 100.00 | | | | | | | | | | | |
| **OsCYP711A2** | 70.42 | 57.14 | 58.05 | 50.48 | 58.61 | 100.00 | | | | | | | | | | |
| **OsCYP711A3** | 71.91 | 60.39 | 59.35 | 53.16 | 63.24 | 81.97 | 100.00 | | | | | | | | | |
| **OsCYP711A4** | 71.64 | 57.20 | 58.43 | 52.79 | 62.33 | 78.62 | 82.53 | 100.00 | | | | | | | | |
| **OsCYP711A5** | 51.76 | 56.42 | 47.94 | 79.82 | 52.17 | 50.67 | 52.60 | 51.55 | 100.00 | | | | | | | |
| **OsCYP711A6** | 57.48 | 71.87 | 53.86 | 54.63 | 59.19 | 58.09 | 58.88 | 58.83 | 55.15 | 100.00 | | | | | | |
| **BdCYP711A5** | 51.57 | 54.71 | 47.08 | 75.00 | 50.51 | 47.75 | 50.88 | 51.37 | 74.02 | 55.35 | 100.00 | | | | | |
| **BdCYP711A6** | 59.77 | 80.98 | 57.42 | 58.06 | 59.11 | 58.25 | 60.38 | 58.58 | 58.05 | 72.31 | 55.90 | 100.00 | | | | |
| **BdCYP711A29** | 59.68 | 53.32 | 73.31 | 49.01 | 56.60 | 57.48 | 59.33 | 59.96 | 47.72 | 52.98 | 46.40 | 55.51 | 100.00 | | | |
| **BdCYP711A30** | 68.48 | 55.66 | 53.37 | 50.10 | 55.70 | 63.40 | 65.50 | 66.29 | 49.16 | 55.58 | 48.57 | 55.68 | 54.79 | 100.00 | | |
| **BdCYP711A31** | 62.50 | 59.15 | 70.91 | 53.74 | 60.47 | 59.47 | 60.70 | 61.00 | 63.20 | 56.80 | 51.16 | 60.15 | 71.59 | 55.30 | 100.00 | |
| **SmCYP711A1** | 39.33 | 39.07 | 38.54 | 37.41 | 39.43 | 38.45 | 39.71 | 38.56 | 36.43 | 37.86 | 35.25 | 38.53 | 38.25 | 36.51 | 38.04 | 100.00 |

**Supplementary Table 5.** ***BdCYP711A29*** **TILLING mutant families** identified following screening of the Bd21-3 TILLING collection (Dalmais et al., 2013). The two mutant families selected for further analysis are indicated in bold letters.

| **Family name** | **Nucleic acid transition** | **Amino acid substitution** | **Type of Mutation** | **SIFT Score** |
|---|---|---|---|---|
| 7758 | G1803A | G421G | Silent | 1 |
| **8687** | **C1831T** | **P431S** | **Missense** | **0** |
| 7708 | G1855A | E439K | Missense | 0 |
| **5374** | **C1888T** | **R450*** | **Stop** | **0** |
| 7424 | C1940A | P467H | Missense | 0.02 |
| 8496 | C1941T | P467P | Silent | 0.06 |
| 7123 | G1944A | G468G | Silent | 1 |
| 6511 | C1972T | L478F | Missense | 0.08 |
| 7846 | C2034T | P498P | Silent | 1 |
| 8480 | C2076T | G512G | Silent | 1 |

**Supplementary Table 6:** Raw data and calculation of relative quantity of oranbanchol in exudates of wild-type (Bd21-3), overexpressing (OE-CYP12.20) and mutant (M5374#135) lines after 7 d phosphorus starvation. AUC: Area under the curve. MRM transitions 321 > 224 and 347 > 97 were used to quantify GR24 and orobanchol signals, respectively.

| **Genotype** | **Bd21-3 (WT)** | | | **OE-CYP12.20** | | | **M5374#135** | | |
|---|---|---|---|---|---|---|---|---|---|
| **Sample** | **Bd21-3 (WT)-A** | **Bd21-3 (WT)-B** | **Bd21-3 (WT)-C** | **OE-CYP12.20-A** | **OE-CYP12.20-B** | **OE-CYP12.20-C** | **M5374 #135-A** | **M5374 #135-B** | **M5374 #135-C** |
| Root FW (g) | 1.0467 | 2.32 | 1.014 | 1.88 | 1.27 | 1.431 | 1.451 | 1.4555 | 1.2059 |
| Orobanchol 347>97 $MH^+$ relative to GR24 321>224 $MH^+$ | 0.091 | 0.130 | 0.122 | 1.854 | 1.629 | 1.566 | 0.058 | 0.123 | 0.06 |
| AUC Orobanchol / AUC GR24 / g root FW | 0.087 | 0.056 | 0.121 | 0.986 | 1.282 | 1.094 | 0.040 | 0.085 | 0.053 |
| **Mean AUC Orobanchol / AUC GR24 / g root FW** | 0.088 | | | 1.121 | | | 0.059 | | |
| **Standard deviation** | 0.032 | | | 0.150 | | | 0.023 | | |

FW: fresh weight
